# Identification of Hypoxia-Related Prognostic Signature and Competing Endogenous RNA Regulatory Axes in Hepatocellular Carcinoma

**DOI:** 10.3390/ijms232113590

**Published:** 2022-11-05

**Authors:** Yulai Tang, Hua Zhang, Lingli Chen, Taomin Zhang, Na Xu, Zunnan Huang

**Affiliations:** 1 The First Dongguan Affiliated Hospital, Guangdong Medical University, Dongguan 523710, China; 2 The First Clinical Medical College, Guangdong Medical University, Dongguan 523808, China; 3 Key Laboratory of Big Data Mining and Precision Drug Design of Guangdong Medical University, Key Laboratory of Computer-Aided Drug Design of Dongguan City, Key Laboratory for Research and Development of Natural Drugs of Guangdong Province, Guangdong Medical University, Dongguan 523808, China; 4 Marine Medical Research Institute of Guangdong Zhanjiang, Zhanjiang 524023, China

**Keywords:** hepatocellular carcinoma, hypoxia, prognosis, competing endogenous RNA, immune

## Abstract

Hepatocellular carcinoma (HCC) is a common type of liver cancer and one of the highly lethal diseases worldwide. Hypoxia plays an important role in the development and prognosis of HCC. This study aimed to construct a new hypoxia-related prognosis signature and investigate its potential ceRNA axes in HCC. RNA profiles and hypoxia genes were downloaded, respectively, from the Cancer Genome Atlas hepatocellular carcinoma database and Gene Set Enrichment Analysis website. Cox regression analyses were performed to select the prognostic genes and construct the risk model. The ENCORI database was applied to build the lncRNA-miRNA–mRNA prognosis-related network. The TIMER and CellMiner databases were employed to analyze the association of gene expression in ceRNA with immune infiltration and drug sensitivity, respectively. Finally, the co-expression analysis was carried out to construct the potential lncRNA/miRNA/mRNA regulatory axes. We obtained a prognostic signature including eight hypoxia genes (ENO2, KDELR3, PFKP, SLC2A1, PGF, PPFIA4, SAP30, and TKTL1) and further established a hypoxia-related prognostic ceRNA network including 17 lncRNAs, six miRNAs, and seven mRNAs for hepatocellular carcinoma. Then, the analysis of immune infiltration and drug sensitivity showed that gene expression in the ceRNA network was significantly correlated with the infiltration abundance of multiple immune cells, the expression level of immune checkpoints, and drug sensitivity. Finally, we identified three ceRNA regulatory axes (SNHG1/miR-101-3p/PPFIA4, SNHG1/miR-101-3p/SAP30, and SNHG1/miR-101-3p/TKTL1) associated with the progression of HCC under hypoxia. Here, we constructed a prognosis gene signature and a ceRNA network related to hypoxia for hepatocellular carcinoma. Among the ceRNA network, six highly expressed lncRNAs (AC005540.1, AC012146.1, AC073529.1, AC090772.3, AC138150.2, AL390728.6) and one highly expressed mRNA (PPFIA4) were the potential biomarkers of hepatocellular carcinoma which we firstly reported. The three predicted hypoxia-related regulatory axes may play a vital role in the progression of hepatocellular carcinoma.

## 1. Introduction

Hepatocellular carcinoma (HCC) accounts for the majority (90%) of primary liver cancers, which is the fourth principal cause of tumor-related death worldwide and has a very high morbidity and mortality [1,2]. Every year, more than 500,000 people worldwide are diagnosed with hepatocellular carcinoma, and about 20,000 new cases occur in the United States [3]. Although there are diverse options for HCC treatment, such as hepatectomy, liver transplantation, radiation, and chemotherapy [4], the overall five-year survival rate is still low (<20%) [5]. Therefore, finding new molecular biomarkers for its diagnosis, survival prognostication, and recurrence monitoring are of great significance for improving the treatment of HCC patients.

Hypoxia is present in a variety of solid tumors that may affect anti-cancer therapy and drive the malignant progression of cancer. Hypoxia is increasingly recognized as a major contributing factor to poor prognosis in cancer therapy [6]. The rapid proliferation of cancer cells consumes a huge amount of oxygen, which destroys the balance of oxygen supply with consumption and forms a hypoxic microenvironment in tumors [7]. Among the vital organs of the human body, the liver is the most vulnerable to hypoxia, which has been demonstrated to be related to metastasis and the poor prognosis of hepatocellular carcinoma [8]. At the same time, immune cells also play a vital role in HCC development [9]. Recent studies have also revealed that hypoxia can regulate the state of the tumor immune microenvironment [10]. However, the potential regulatory mechanisms in hypoxia, immunity and hepatocellular carcinoma remain unclear. Therefore, further studies are needed on the relationship of hypoxia with immunity in hepatocellular carcinoma.

In 2011, Salmena et al. suggested the concept of the competing endogenous RNA (ceRNA), which was defined as a class of RNA to regulate the transcription of other RNAs by competitively binding to the shared microRNA (miRNA) [11]. LncRNA competitively binds to miRNA, regulates the mRNA expression level, and affects the translation of corresponding proteins and related cell activities. The interaction between them in the progression and prognosis of HCC had been verified by considerable computer research and experimental studies [12,13]. More and more studies demonstrated that the ceRNA gene interaction network played an important role in the tumorigenesis, progression and prognosis of HCC. For example, Li et al. demonstrated that up-regulation of lnc-APUE inhibited the expression of miR-20b in hepatocellular carcinoma, leading to an increase in E2F1 level, thus accelerating G1/S transformation and tumor cell proliferation [14]. Hu et al. showed that overexpression of lincSCRG1 restricted the ability of miR26a to derepressing the upregulated SKP2, thus inducing the proliferation and migration of HCC cells [15]. Although bio-scientists currently have a certain understanding of the lncRNA-related ceRNA network in hepatocellular carcinoma, the studies on the HCC hypoxia-related ceRNA regulatory network remain unreported. Therefore, exploring the hypoxia-related ceRNA regulatory network and identifying its key regulatory axes and nodes may provide new candidate therapeutic targets for the treatment of hepatocellular carcinoma.

**Figure 1:**
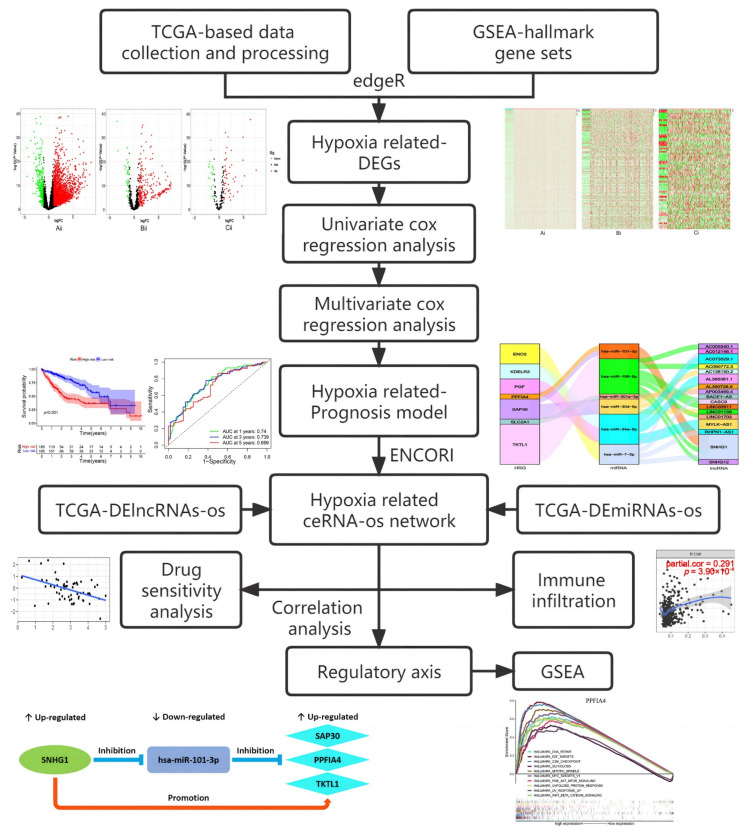
The workflow of this study: Analysis of hypoxia-related prognostic biomarkers and key axes. The diagram contains specific bioinformatics approaches, data mining tools, and partial analysis results.

With the development of bioinformatics, the molecular pathogenesis of tumors analyzed by bioinformatics has opened up an important path for tumor research. In this study, we extracted differentially expressed lncRNAs (DE-lncRNAs), miRNAs (DE-miRNAs) and hypoxia-related differentially expressed mRNAs (HR-DEmRNAs), respectively, from HCC data stored in The Cancer Genome Atlas (TCGA) database and the Hallmark dataset stored in the Gene Set Enrichment Analysis (GSEA) website. Then, we identified the prognostic-related RNAs by univariate Cox regression analysis and further established a hypoxia gene prognosis signature by multivariate Cox regression analysis. After that, we performed targeting pairing of the model genes in ENCORI database, and constructed a hypoxia-related prognosis ceRNA network. The relationship with mRNA expression in the ceRNA network of immune infiltration, immune biomarker and drug sensitivity was further analyzed by TIMER, GEPIA and CellMiner databases, respectively. Finally, we identified important potential regulatory axes based on the targeted regulations of “lncRNA/miRNA/mRNA” in the ceRNA network, and explored their potential role as the regulatory axes in hepatocellular carcinoma by GSEA (Figure 1). This study can provide new candidate targets for the HCC poor prognosis and help to better understand the regulatory mechanism of ceRNAs in the hypoxia environment on the occurrence and development of hepatocellular carcinoma.

## 2. Results

### 2.1. Screening of Differentially Expressed Genes

**Figure 2:**
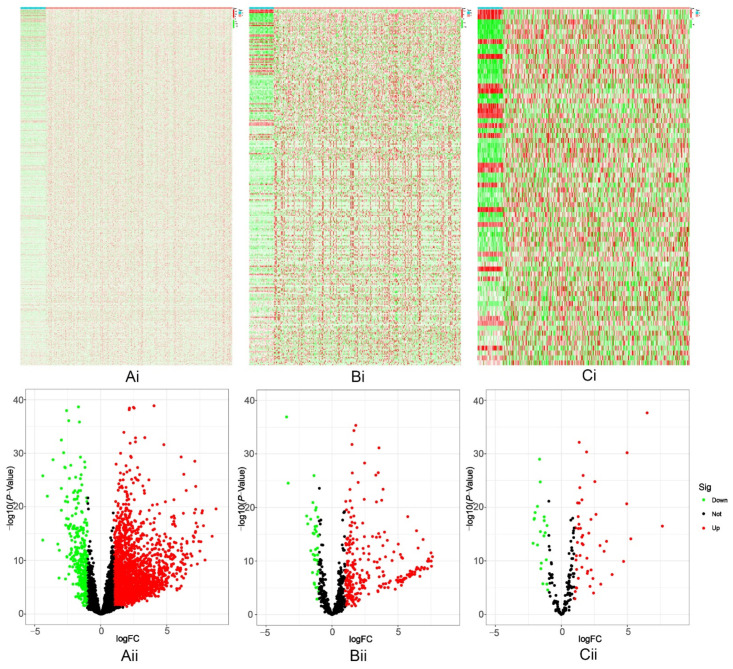
Identification of differentially expressed lncRNAs, miRNAs, and hypoxia-related mRNAs in human hepatocellular carcinoma. (**Ai**–**Ci**): Heatmap. (**Aii**–**Cii**): Volcano plot. The red/green dots denote statistically significantly up-/down-regulated lncRNAs, miRNAs, and hypoxia-related mRNAs, respectively. The black dots denote non-differentially expressed RNAs.

A total of 2553 (upregulated: 2211, downregulated: 342) DE-lncRNAs (Figure 2(Ai,Aii), Table S1), 300 (upregulated: 260, downregulated: 40) DE-miRNAs (Figure 2(Bi,Bii), Table S2), and 72 HR-DEmRNAs (upregulated: 50, downregulated: 22) (Figure 2(Ci,Cii), Table S3) were identified from hepatocellular carcinoma samples from the TCGA.

### 2.2. Screening of Prognosis-Related RNAs

By using univariate Cox analysis, a total of 690 prognosis-related RNAs were screened (612 lncRNAs, 53 miRNAs, and 25 mRNAs). Among them, 545 DE-lncRNAs-os were upregulated and 67 DE-lncRNAs-os were downregulated (Table S4); 40 DEmiRNAs-os were upregulated and 13 DE-miRNAs-os were downregulated (Table S5); 19 HR-DEmRNAs-os were upregulated and 6 HR-DEmRNAs-os were downregulated (Table S6).

### 2.3. Construction of a Prognostic Hypoxia-Related Gene Signature 

**Figure 3:**
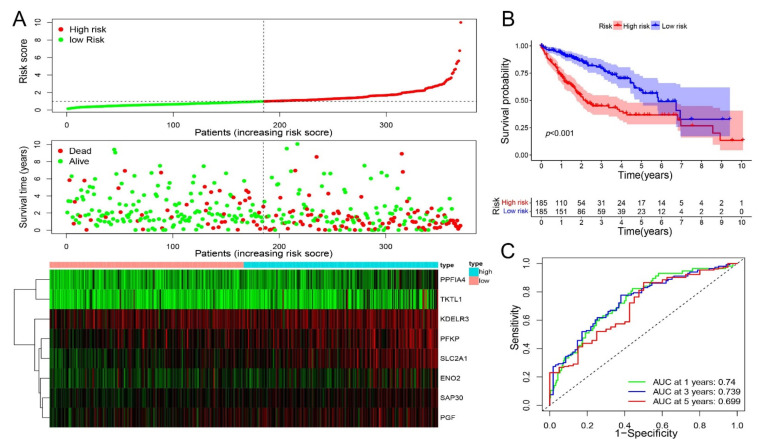
Prognostic risk scoring model of eight hypoxia-related genes in hepatocellular carcinoma patients. (**A**). Risk score distribution of HCC patients, survival status distribution of HCC patients, heatmap of eight mRNA expression profile ranked by the risk score. (**B**). Kaplan-Meier curve for low-risk and high-risk subgroups by the risk score. (**C**). Time-dependent ROC curves for forecasting survival in HCC patients by the risk score.

A hypoxia-related signature for prognosis was established from multivariate Cox regression analysis, which consisted of eight genes from HR-DEmRNAs-os. The risk score was calculated as follows: risk score = (−0.258850669×ENO2 exp) + (0.111022896×KDELR3 exp) + (0.121807561×PFKP exp) + (0.199403905×PGF exp) + (0.129762047×PPFIA4 exp) + (0.36354863×SAP30 exp) + (0.212227539×SLC2A1 exp) + (0.05656501×TKTL1 exp). According to the median value of risk score, the hepatocellular carcinoma patients were divided into a low-risk subgroup and a high-risk subgroup (Figure 3A). There were statistically significant variations in survival time between the low-risk and high-risk subgroups (Figure 3B). The AUC values for Overall Survival (OS) predicted by this model at 1 year, 3 years, and 5 years were 0.740, 0.739, and 0.699, respectively, indicating that this model had a moderate discriminative ability (Figure 3C).

### 2.4. Construction of the Hypoxia-Related ceRNA Network 

**Figure 4:**
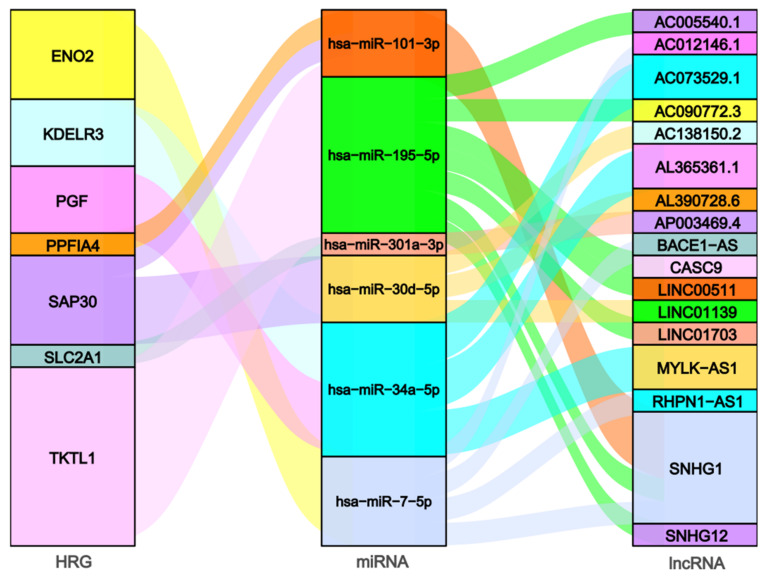
LncRNA-miRNA-mRNA network for hypoxia-related prognosis of hepatocellular carcinoma.

A total of six prognosis-related DEmiRNA-os and 17 prognosis-related DE-lncRNA-os were screened out from the ENCORI database based on the prognostic model-related seven HR-DEmiRNA-os (ENO2, KDELR3, PGF, PPFIA4, SAP30, SLC2A1, and TKTL1) (Table 1) and it is worth to note that non-eligible miRNA-mRNA pairs were screened out for HR-DEmiRNA-os PFKP. The miRNA-mRNA pairs (Table S7) and miRNA-lncRNA pairs (Table S8) among these HR-DEmRNA-os, DEmiRNA-os, and DE-lncRNA-os formed a hypoxia-related prognostic ceRNA network as shown in Figure 4.

### 2.5. Correlation between ceRNA Network and Tumor Immune Infiltration

**Figure 5:**
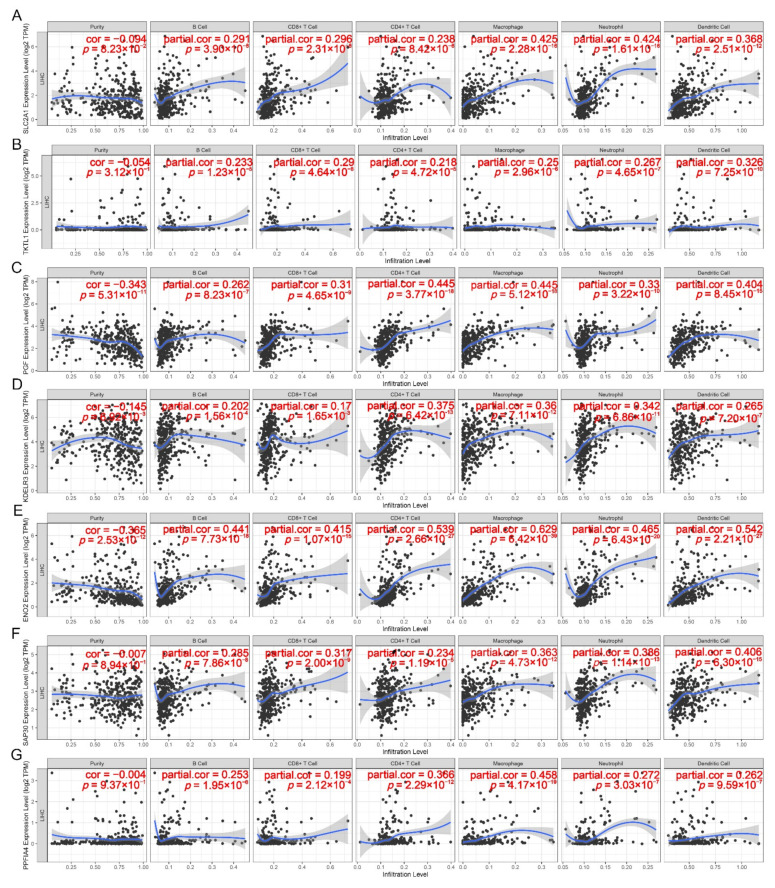
Correlation between the expressions of seven HR-DEmRNA-os and six tumor immune cells in hepatocellular carcinoma (*p* < 0.05 was considered to be significantly correlated). (**A**). SLC2A1, (**B**). TKTL1, (**C**). PGF, (**D**). KDELR3, (**E**). ENO2, (**F**). SAP30, (**G**). PPFIA4.

The TIMER analysis showed that the expressions of seven HR-DEmRNA-os in the hypoxia-related ceRNA network were statistically significantly associated with six infiltrating immune cells in the human hepatocyte microenvironment (Figure 5A–G). Among them, ENO2 (cor = 0.629), PPFIA4 (cor = 0.458), and SLC2A1 (cor = 0.425) had the highest correlation with macrophages; KDELR3 (cor = 0.375) had the highest correlation with CD4+T cells; PGF had the highest correlation with CD4+T cells (cor = 0.445) and macrophages (cor = 0.445); SAP30 (cor = 0.406) and TKTL1 (cor = 0.326) had the highest correlation with dendritic cells. On the other side, macrophages (cor = 0.629), dendritic cells (cor = 0.542), CD4+T cells (cor = 0.539), Neutrophils (cor = 0.465), B cells (cor = 0.441), and CD8+T cells (cor = 0.415) all had the highest correlation with ENO2, indicating that this gene, among seven HR-DEmRNA-os in the ceRNA network, might play the most important role in tumor immune infiltration. 

The positive correlation between the hypoxia-associated ceRNA and immune infiltration could be also verified via the TIMER analysis on the relationship of seven HR-DEmRNA-os with 21 biomarkers in these immune cells. The results showed that they were statistically significantly associated with each other in the vast majority of cases (*p* < 0.05, Table S9). In addition, each of these HR-DEmRNA-os except for TKTL1, had a cor > 0.3 with several biomarkers of immune cells. Especially, ENO2 had a correlation coefficient of more than 0.3, 0.4 and 0.5, respectively, with the 19, 16, and 5 out of 21 biomarkers, and had the highest correlation with ITGAX (R = 0.670) in dendritic cells, again supporting its potential vital role on tumor immune infiltration. 

The correlation analysis from TIMER further showed a positive correlation with the statistical significance between the expressions of HR-DEmRNA-os and three immune checkpoints (*p* < 0.05, Table S10). Among them, PGF (cor = 0.398), PPFIA4 (cor = 0.315) and TKTL1 (cor = 0.228) had the highest correlation with PDCD1; SAP30 (cor = 0.377) and SLC2A1 (cor = 0.361) had the highest correlation with CD274; ENO2 (cor = 0.562) and KDELR3 (cor = 0.283) had the highest correlation with CTLA-4. It is worth noting that on the whole ENO2 showed the strongest correlation with these immune checkpoints as it also had a correlation coefficient of 0.560 and 0.343, respectively, with PDCD1 and CD274. 

The correlation analyses were also performed in the GEPIA database and similar results were obtained on the positive correlation between the expressions of seven HR-DEmRNA-os and those of 21 immune cell markers as well as three immune checkpoints (Tables S9 and S10). All the above results suggested a statistically significant correlation of these HR-DEmRNA-os with tumor immune infiltration and thus this ceRNA regulatory network might take an important part in HCC immune escape under a hypoxia microenvironment.

### 2.6. Drug Sensitivity Analysis

Table S11 shows 110 correlation pairs between the abnormally up-regulated expressions of seven HR-DEmRNA-os in the hypoxia-associated ceRNA network and drug susceptibility of 82 FDA-approved antitumor drugs in NCI-60 cancer cell line data. Among these drugs, 64 drugs with the formation of 80 pairs were shown to decrease the drug sensitivity with the upregulation of hypoxia-related mRNAs, while 14 drugs with the formation of 22 pairs behaved oppositely. In addition, four drugs with the formation of eight pairs exhibited the change of their drug sensitivities oppositely with the different upregulated hypoxia genes. Specifically, the high expression of ENO2 was associated with reduced drug sensitivity of cancer cells to 12 drugs (Docetaxel, Paclitaxel, Eribulin mesylate, Allopurinol, Vinorelbine et al.) and with increased drug sensitivity of cancer cells to one drug (Midostaurin). The high expression of KDELR3 was associated with reduced drug sensitivity of cancer cells to 53 drugs (Valrubicin, Cyclophosphamide, Teniposide, Palbociclib, Daunorubicin, Oxaliplatin et al.) and with increased drug sensitivity of cancer cells to eight drugs (Irofulven, Zoledronate, Simvastatin, Abiraterone, IPI-145 et al.). The high expression of PGF was associated with reduced drug sensitivity of cancer cells to four drugs (Gefitinib, Palbociclib, Lapatinib, and Allopurinol), and with increased drug sensitivity of cancer cells to another four drugs (Vemurafenib, Irofulven, ARRY-162, and Encorafenib). The high expression of PPFIA4 was associated with reduced drug sensitivity of cancer cells to one drug (Dexrazoxane) and with increased drug sensitivity of cancer cells to three drugs (Idelalisib, Abiraterone, and IPI-145). The high expression of SAP30 was associated with reduced drug sensitivity of cancer cells to one drug (Erlotinib) and with increased drug sensitivity of cancer cells to five drugs (Ifosfamide, Nelarabine, Carmustine, Lomustine, and Arsenic trioxide). The high expression of SLC2A1 was associated with reduced drug sensitivity of cancer cells to six drugs (Denileukin Diftitox Ontak, DIGOXIN, Arsenic trioxide, Bendamustine, Bortezomib, and Ixazomib citrate), and with increased drug sensitivity of cancer cells to four drugs (Simvastatin, Irofulven, IPI-145, and Dasatinib). The high expression of TKTL1 was associated with reduced drug sensitivity of cancer cells to seven drugs (6-Thioguanine, Parthenolide, Allopurinol, 6-Mercaptopurine, and Pazopanib), and with increased drug sensitivity of cancer cells to one drug (TYROTHRICIN). The above results showed a complex correlation between hypoxia-related mRNAs and drug susceptibility, which might provide more options for chemotherapy in HCC patients. 

### 2.7. Identification of Potential lncRNA-miRNA-mRNA ceRNA Regulatory Axes

**Figure 6:**
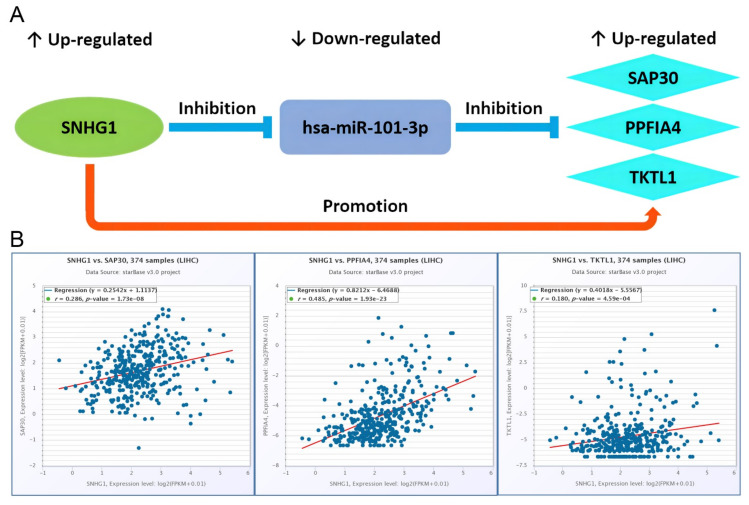
Recognition of three potential lncRNA-miRNA-mRNA ceRNA regulatory axes. (**A**). The targeted regulation relationship among the lncRNA, miRNA, mRNA of the three potential ceRNA regulatory axes in the HCC development. (**B**). The correlation (*p* < 0.05 was considered as to be significantly correlated) of SNHG1 with PPFIA4, SAP30, and TKTL1, respectively.

According to the regulatory relationships of RNAs (mRNA upregulated, miRNA downregulated, mRNA upregulated, as shown in Table 1) in the ceRNA network (Figure 4), the seven DElncRNA-os and two DEmiRNA-os listed in Table S8 formed eight DE-lncRNA-os--DE-miRNA-os pairs via the correlation analysis of the ENCORI database (Figure S1). Among them, six showed statistically significantly negative correlations (*p* < 0.05) with SNHG1--hsa-miR-101-3p pair being most (*p* = 1.42 × 10^−12^) while two did not (Table S12). Likewise, the two DEmiRNA-os and three DEmRNA-os listed in Table S7 formed four DE-miRNA-os--DE-mRNA-os pairs (Figure S1). Three pairs of them showed a statistically significant negative correlation between hsa-miR-101-3p and PPFIA4, SAP30, and TKTL1 (*p* < 0.05), respectively, while one did not (Table S13). With DEmiRNA-os as the connection point, we combined six DE-lncRNA-os--DE-miRNA-os pairs and three DE-miRNA-os--DE-mRNA-os pairs to obtain three potential lncRNA/miRNA/mRNA ceRNA regulatory axes (SNHG1/hsa-miR-101-3p/SAP30, SNHG1/hsa-miR-101-3p/PPFIA4, SNHG1/has-miR-101-3p/TKTL1) (Figure 6A). In addition, among the indirect DE-lncRNA-os and HR-mRNA-os pairs, the correlation as positive as needed between lncRNA SNHG1 and PPFIA4, between SNHG1 and SAP30, between SNHG1 and TKTL1 was 0.485 (*p* = 1.93 × 10^−23^), 0.286 (*p* = 1.73 × 10^−8^), 0.180 (*p* = 4.59 × 10^−4^), respectively, all of which showed statistically significant (Figure 6B). 

### 2.8. Gene Set Enrichment Analysis

**Figure 7:**
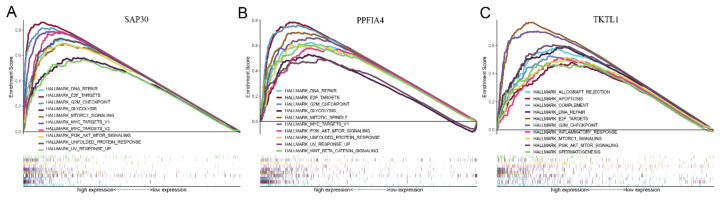
GSEA enrichment analysis of three hypoxia-related mRNAs in the predicted ceRNA regulatory axes. (**A**) SAP30, (**B**) PPFIA4, (**C**) TKTL1.

The GSEA analysis showed that the highly expressed PPFIA4, SAP30, and TKTL1 in the ceRNA regulatory axes were all significantly enriched in a variety of hypoxia-and cancer-related pathways (Figure 7A–C), such as DNA repair, E2F target gene, G2/M checkpoint and PI3K/AKT/mTOR pathway, indicating that hypoxia-related genes PPFIA4, SAP30, TKTL1 were closely related with tumor microenvironment and HCC development.

## 3. Discussion

The progression of hepatocellular carcinoma is a complex process affected by a variety of factors. Tumor hypoxia is one of the adverse factors for cancer treatment, and it participates in HCC occurrence and significantly increases HCC invasiveness [7]. LncRNA can act on miRNA to further target and regulate mRNA, all of which form the lncRNA/miRNA/mRNA regulatory axis, and then participate in the occurrence, development and metastasis of hepatocellular carcinoma [16,17]. The construction of the ceRNA networks under hypoxia is helpful for us to comprehend the molecular mechanism of hypoxia-related genes in hepatocellular carcinoma and find new prognostic markers and treatment entry points for hepatocellular carcinoma. 

Our study is based on hepatocellular carcinoma RNA profiles from the TCGA database and hypoxia-related genes from the GSEA website. By using differential expression analyses through R language, we identified the expression matrices of DE-lncRNAs, DE-miRNAs and hypoxia-related mRNAs (HR-DEmRNAs). A total of 690 prognosis-related RNAs (612 DE-lncRNA-os, 53 DE-miRNA-os, and 25 HR-DEmRNAs-os) were screened out by univariate Cox regression analysis. After that, a hypoxia-related prognosis signature consisting of eight genes (ENO2, KDELR3, PFKP, SLC2A1, PGF, PPFIA4, SAP30, and TKTL1) was constructed by multivariate Cox regression analysis.

Table 2 gave a comparative analysis of the abnormal expression of eight prognostic model-related hypoxia genes in hepatocellular carcinoma with the existing literature reports. Among the eight mRNAs in the model, the up-expressions of TKTL1, KDELR3, PFKP, SLC2A1, and PGF in hepatocellular carcinoma were confirmed in previous experimental studies, while the over-expression of SAP30 in HCC was reported in an earlier computational study. In this study, we first proposed that the abnormally high expressions of ENO2 and PPFIA4 was related to the poor prognosis of hepatocellular carcinoma, though they had been experimentally reported in other cancers. The high expressions of SAP30, ENO2, and PPFIA4 in HCC, especially under hypoxia, need to be confirmed on the laboratory by future studies.

Based on the model genes, a hypoxia-related prognostic ceRNA network consisting of 17 lncRNAs, six miRNAs, and seven mRNA was constructed. Table 3 showed a comparative analysis of the abnormal expressions of the six miRNAs in hepatocellular carcinoma between this study and the existing literature. The abnormal expressions of five miRNAs (three upregulated: hsa-miR-301a-3p, hsa-miR-30d-5p, hsa-miR-7-5p; two downregulated: hsa-miR-195-5p, hsa-miR-101-3p) were confirmed in previous experimental studies of HCC without mentioning under hypoxia. Interestingly, Ahmed et al. [30] experimentally reported that the expression of hsa-miR-34a-5p was downregulated in HCC; however, Wan et al. reported in their laboratory study that miR-34a-5p was upregulated under in HCC oxidative stress conditions in consistence with our calculated result [31], indicating the potentially different functions of this miRNA on the occurrence and development of HCC with or without hypoxia. As a result, the abnormal expressions of the ceRNA network-related six miRNAs under conditions of hypoxia in HCC deserved further investigation.

Table 4 gave a comparative analysis of the abnormal expression of these 17 lncRNAs in hepatocellular carcinoma with the existing literature reports. Among the 17 lncRNA, only AL365361.1 was calculated as downregulated and the remaining 16 lncRNAs were shown as upregulated in HCC. The upregulations of eight lncRNAs (BACE1-AS, CASC9, LINC00511, LINC01139, MYLK-AS1, RHPN1-AS1, SNHG1, and SNHG12) were confirmed in previous experimental studies. In addition, although three lncRNAs (LINC01703, AL365361.1, and AP003469.4) with abnormal expressions in HCC had not been demonstrated by experimentation earlier, the over-expression of LINC01703 was experimentally reported in non-small cell lung cancer (NSCLC), and the over-expressed AP003469.4 and the down-expressed AL365361.1 were computationally reported in HCC, which were also in accord with our calculated results. The remaining six highly expressed lncRNAs (AC005540.1, AC012146.1, AC073529.1, AC090772.3, AC138150.2, and AL390728.6), which were shown to be related to the poor prognosis of HCC in this study, have not been reported in any cancer studies before. Thus, these six lncRNAs were discovered here to be the potential biomarkers in HCC for the first time. The abnormal expressions of the latter nine lncRNAs in HCC need to be experimentally confirmed in future studies.

Hypoxia is an important component of tumor microenvironment in solid tumors [49]. It is crucial to understand the effects of hypoxia genes on the molecular mechanisms of cancer for improving cancer treatment [6,50]. The characteristics of hypoxia-related genes can make them become reliable HCC biomarkers for predicting the cancer prognosis and therapeutic response. Further research on the molecular mechanism of ceRNA regulatory axes in cancer occurrence and development based on hypoxia-related genes can also be very significant. At present, there have been some experimental studies on the ceRNA mechanisms related to cancer and hypoxia. For example, Zhang et al. showed that hypoxia-induced transcriptional upregulation of lncRNA-NEAT1 in human hepatoma cell lines (SNU-182 and HUH7), which further promoted the progression of HCC cells via the lncRNA-NEAT1/miR-199a-3p/UCK2 pathway [51]. Hu et al. revealed that hypoxia upregulated the expression of lncHILAR (LOC100506178) in human renal cell carcinoma (RCC) cell lines (Caki-1 and ACHN), which further promoted the invasion and metastasis of RCC cells through the mir-613/206/1-1-3p/jagged-1/notch/CXCR4 ceRNA axis [52]. Experimental studies like this revealed that hypoxia could promote the biological process of some cancers via key regulatory axes and nodes in the hypoxia-related ceRNA network. Likewise, the ceRNA regulatory axes we constructed here based on hypoxia-related genes may complement the underlying mechanisms of HCC progression under hypoxia and provide new perspectives and candidate therapeutic targets for the treatment of hepatocellular carcinoma.

The abundance of tumor immune cell infiltration and the over-regulation of immune checkpoints were closely linked to the prognosis of cancer patients [53,54], whose exploration is of great impact on improving the efficacy of immunotherapy for hepatocellular carcinoma [55,56]. In this study, we also evaluated their relationship with the expressions of the mRNAs in the hypoxia-related prognostic ceRNA network. The results showed that the expressions of those hypoxia-related genes were significantly correlated with immune cells, including neutrophils, macrophages, dendritic cells, B cells, CD4+T cells, and CD8+T cells in HCC, indicating that upregulating the hypoxia-related mRNAs could improve the infiltration of tumor immune cells. In addition, the effect of immunotherapy is not only related to the infiltration of immune cells in the cancer microenvironment, but also depended on the expression of immune checkpoints [57]. We found that the hypoxia-related mRNAs, especially ENO2, in the ceRNA network were positively associated with PD1, PD-L1, or CTLA-4 in HCC, indicating that upregulating these mRNAs could promote the immune escape of cancer cells in hepatocytes, and targeting them could increase the efficacy of immunotherapy of patients with hepatocellular carcinoma.

By using CellMiner, we screened out 82 FDA-approved antitumor drugs based on the abnormal expressions of hypoxia-related mRNAs in the ceRNA network and analyzed their drug sensitivity that might provide new perspectives for the clinical treatment of HCC patients. Partial drugs have already been demonstrated to have a therapeutic effect on HCC via the different mechanisms by experimentation in previous studies. For examples, Feng et al. showed that Simvastatin inhibited the HIF-1α/PPAR-γ/PKM2 axis, and further suppressed PKM2-mediated glycolysis, causing reduced proliferation and enhanced apoptosis in hepatocellular carcinoma cells [58]. Xiang et al. showed that Cabozantinib inhibited the VEGFR2 and MET signaling pathway to prevent the tumor growth, angiogenesis, and metastasis of HCC cells [59]. Chang et al. showed that Dasatinib inhibited the SFK/FAK and PI3K/PTEN/Akt pathways by down-regulating the Src tyrosine kinase, thus preventing the adhesion, proliferation, invasion, and migration of HCC cells in vitro [60]. Yue et al. showed that Idelalisib inhibited AKT, activated FoxO3a, and further upregulated Bim, thus promoting the development of mitochondria-dependent apoptosis in HCC cells [61]. However, many other drugs including Abiraterone, ARRY-162, Carmustine, Encorafenib, IPI-145, Irofulven, Midostaurin, TYROTHRICIN, and Vemurafeniba, etc., which might also have the therapeutic effect on HCC have not been reported in the earlier studies. Thus, further research is required to explore the therapeutic effect and mechanisms of these drugs against HCC. Moreover, among these FDA drugs, 64 showed to have drug sensitivity decreased with the upregulation of hypoxia-related mRNAs and 14 drugs acted oppositely when considering the reaction of cancer cells. Because these results could help the physician to prescribe more-sensitivity drugs to individual HCC patients with different abnormal expressions of the prognostic genes, the drug sensitivity analysis is of huge significance for the precision treatment of HCC patients. Thus, this study may provide a new perspective on the development of precision medicine and its results might play an important role in improving the efficacy of chemotherapy for the medical treatment of HCC patients.

Through RNA correlation analysis, we identified three lncRNA/miRNA/mRNA regulatory axes (SNHG1/hsa-miR-101-3p/SAP30, SNHG1/hsa-miR-101-3p/PPFIA4, SNHG1/hsa-miR-101-3p/TKTL1) that might be involved in the HCC development under the hypoxia environment. Up to now, no experimental reports have confirmed these three ceRNA regulatory axes. However, the abnormal expressions of these axis-related RNAs were demonstrated in previous cancer studies as follows. The over-expression of lncRNA-SNHG1 [43] and mRNA-TKTL1 [21], and the down-expression of miRNA-hsa-miR-101-3p [36] were confirmed in various experimental studies of hepatocellular carcinoma, respectively. In addition, the up-expressions of SAP30 and PPFIA4 were also, respectively, verified in a bioinformatics study of hepatocellular carcinoma [27] and an experimental study of colorectal carcinoma [29]. What is more, Wei et al. showed by experimentation that the highly expressed lncRNA-SNHG1 could directly target hsa-miR-101-3p (which form the basis of the three ceRNA axes we predicted for HCC) to up-regulate ROCK2, and thus lowering the sensitivity of NSCLC cells to cisplatin in non-small cell lung cancer tissues [62]. Therefore, we speculated that these three hypoxia-related ceRNA regulatory axes were reliable and significant in the malignant biological process of HCC, which could provide a direction for studying the pathological mechanism and serve as candidate targets for clinical diagnosis, treatment and prognosis of hepatocellular carcinoma. However, their specific signaling pathways and function mechanisms need to be further investigated in future experimental studies.

## 4. Materials and Methods

### 4.1. Data Download and Differential Expression Analysis

The clinical data and expression profiles of hepatocellular carcinoma were downloaded from the TCGA database (https://portal.gdc.cancer.gov/ (accessed on 9 August 2021)). The data mainly included the following three aspects: (1) RNA sequencing (RNA-seq):424 (normal: 50, tumor: 374) lncRNA and 424 (normal: 50, tumor: 374) mRNA cases; (2) miRNA sequencing (miRNA expression): 425 (normal: 50, tumor: 375) miRNA cases; (3) clinical survival time of the cases. 

Two hundred hypoxia-related genes, defined as the genes upregulated in response to low oxygen levels (hypoxia) [63], were downloaded from Hallmark Gene Sets on the GSEA website (http://www.gsea-msigdb.org/gsea/index.jsp (accessed on 9 August 2021)). GSEA database includes a collection of “hallmark” gene sets derived from Molecular Signatures Database (MSigDB) [63]. MSigDB developed those collections by a combination of automated methods and expert curation, which assessed the statistically significant information coefficient of all genes, produced nominal *p*-values to create an empirical null distribution by using a sample permutation test and further calculated False Discovery Rates (FDR) based on summary *p*-values [63]. The genes in the original hallmark from the literature were sorted according to their FDR values, the top of which was selected to form the final hallmark set with summary FDR values less than 0.01 [63]. However, when the number of genes obtained by the above steps was less than 15 (or more than 200), selecting the top scoring 15 (or 200) genes ignored whether the summary FDR values of these genes were less than 0.01 or not [63]. Thus, it is the default setting that the hallmarks consist of at least 15 and at most 200 genes in GSEA.

With the help of R software (Version 4.1.2, https://www.r-project.org/ (accessed on 9 August 2021)) and the R package “edgeR”, “ggplots”, the differential expression of lncRNAs (DE-lncRNAs), miRNAs (DE-miRNAs) and the differential expression of hypoxia mRNAs (HR-DEmRNAs) were screened out and drawn, respectively, with the *p*-adj < 0.05, |log_2_FC| > 1 as the screening condition. 

### 4.2. Screening of Prognosis-Related RNA by Univariate Cox Regression Analysis

Univariate Cox regression analysis was carried out using the R package “survival” for DElncRNAs, DEmiRNAs, and HR-DEmRNAs, respectively. The *p* value < 0.05 was used as the criterion to screen out the prognosis-related DE-lncRNAs (DE-lncRNAs-os), DE-miRNAs (DE-miRNAs-os) and HR-DEmRNAs (HR-DEmRNAs-os).

### 4.3. Construction of Hypoxia-Related Gene Prognosis Signature by Multivariate Cox Regression Analysis

Multivariate Cox regression analysis was carried out to construct the hypoxia-related gene prognosis model using HR-DEmRNAs-os. The risk score of the patients was calculated according to the expression level and the correlation coefficients of the model gene as follows: Risk score = Exp (mRNA1) × β1 + Exp (mRNA2) × β2 + Exp (mRNA3) × β3... + Exp (mRNAn) × βn. The patients were divided into a high-risk group and a low-risk group through the median value of the risk score, and the survival rates of the two groups were calculated. Finally, the risk curve was drawn using the R package “pheatmap”, and the ROC curve was drawn utilizing the R package “survivalROC” to evaluate the reliability of the prognosis signature.

### 4.4. Construction of Hypoxia-Related ceRNA Network

The Encyclopedia of RNA Interactomes (ENCORI, http://starbase.sysu.edu.cn/ (accessed on 31 August 2021)) is an openly licensed and state-of-the-art platform to analyze the cellular interaction networks among various RNAs [64]. The ENCORI website was used to extract prognosis-related miRNA-lncRNA pairs and miRNA-mRNA pairs based on HR-DEmRNAs-os in the hypoxia-related gene prognosis signature. The mRNA upstream binding miRNAs were selected according to the following criterium: they were screened out at least in two of seven databases (microT, miRmap, miRanda, PicTar, PITA, RNA22, and TargetScan), while the miRNA upstream binding lncRNAs were just screened out in miRanda database. All of these eight databases were integrated into the ENCORI database [64]. The mutually paired DE-lncRNAs-os, DE-miRNAs-os, and the prognosis model-related HR-DEmRNAs-os were then used as nodes to construct the hypoxia-related prognosis lncRNA-miRNA-mRNA network, which was visualized using the R package “ggalluvial”. 

### 4.5. Correlation Analysis between Hypoxic ceRNA Network and Tumor Immune Infiltration

TIMER web server (https://cistrome.shinyapps.io/timer/ (accessed on 25 September 2022)) is a comprehensive resource for systematical analysis of immune infiltrates on 32 diverse cancer types based on 10,897 tumor samples stored in TCGA and provides six main analysis modules (Gene, Survival, Mutation, SCNA, Diff Exp, Correlation) for users to assess the association between immune infiltration and various factors (such as gene expression, clinical outcomes, somatic mutations, and somatic copy number alterations, etc.) [65]. Here the “Gene” function module was utilized to estimate the correlation between HR-DEmRNAs-os expression in the hypoxia-related ceRNA regulatory network and the level of immune invasion of human hepatocellular carcinoma [65]. The “Correlation” function module was used to evaluate the expression relationship of HR-DEmRNAs-os with immune cell biomarkers and immune checkpoints in hepatocellular carcinoma [65]. 

The GEPIA database (http://gepia.cancer-pku.cn/ (accessed on 25 September 2022)) is an online site for gene expression analysis on 33 diverse cancer types based on 9736 tumors and 8587 normal samples from the TCGA and GTEx (https://www.gtexportal.org/home/datasets (accessed on 25 September 2022)) databases and provides seven major features (General, Differential Genes, Expression DIY, Survival, Similar Genes, Correlation, and PCA) for users to perform customizable profiling plotting, differential expression analysis, patient survival analysis and so on [66]. Here the “Correlation” feature was used to evaluate the expression relationship of HR-DEmRNAs-os in the hypoxia-related ceRNA regulatory network with immune cell markers andimmune checkpoints in hepatocellular carcinoma [66]. 

In both databases, the Spearman coefficient was used to estimate the correlation of hypoxic ceRNA genes with tumor immune infiltration and *p* < 0.05 was set as the selection criterion with statistical significance. 

### 4.6. Drug Sensitivity Analysis

Drug-related data approved by the FDA were downloaded from the CellMiner database (https://discover.nci.nih.gov/cellminer/ (accessed on 12 April 2022)) [67] and the relationship between mRNA and compound susceptibility in hypoxia-associated ceRNA was evaluated using Pearson correlation analysis by the NCI-60 analytical tool. The R packages “limma” and “ggplot2” were used to draw correlation diagrams. The scatter plots were sorted according to the *p* value from small to large, and the *p* < 0.05 was taken as the selection criterion for identifying statistical significance.

### 4.7. Construction of lncRNA/miRNA/mRNA Regulatory Axes

Based on the ceRNA hypothesis, a lncRNA should be negatively associated with miRNA expression and positively associated with the mRNA level. LncRNA downregulates the expression level of miRNA to reduce its inhibitory effect on mRNA. The ENCORI database was applied to analyze the correlation of the above three types of RNAs in the ceRNA network, and screened out the ceRNA regulatory axis and *p* < 0.05 was considered to be statistically significant.

### 4.8. Functional Enrichment Analysis

In order to further elucidate the potential roles of mRNAs in the LncRNA/miRNA/mRNA regulatory axes during the human HCC process, functional enrichment analysis of these ceRNA regulations was performed using the gene set “h.all.v7.4.symbols.gmt [Hallmarks]” as a reference in GSEA database. The genome alignment was tested for 1000 times and the gene set with a nominal *p* < 0.05 was considered to be statistically significant.

## 5. Conclusions

In summary, we constructed a prognostic hypoxia-related ceRNA network for hepatocellular carcinoma via bioinformatics analyses, in which six highly expressed lncRNAs (AC005540.1, AC012146.1, AC073529.1, AC090772.3, AC138150.2, AL390728.6) and one highly expressed mRNA (PPFIA4) were discovered for the first time in this study to be the potential biomarkers of hepatocellular carcinoma. In addition, we identified three ceRNA regulatory axes (SNHG1/miR-101-3p/PPFIA4, SNHG1/miR-101-3p/SAP30 and SNHG1/miR-101-3p/TKTL1), which might play an essential role in HCC progression. 

## Figures and Tables

**Table 1:** Seven HR-DEmRNA-os, six DEmiRNA-os, and 17 DE-lncRNA-os in a hypoxia-related prognostic ceRNA network.

RNA	Expression Change	Name	*p*-adj.
lncRNA	Upregulated	MYLK-AS1	5.61 × 10^−25^
		SNHG1	2.77 × 10^−22^
		BACE1-AS	7.27 × 10^−20^
		LINC01703	9.28 × 10^−18^
		LINC00511	4.58 × 10^−14^
		SNHG12	1.48 × 10^−13^
		AP003469.4	6.04 × 10^−13^
		AC012146.1	7.44 × 10^−12^
		AL390728.6	7.55 × 10^−11^
		RHPN1-AS1	5.45 × 10^−10^
		AC073529.1	4.16 × 10^−9^
		AC090772.3	1.87 × 10^−7^
		LINC01139	5.27 × 10^−7^
		CASC9	1.98 × 10^−6^
		AC138150.2	3.77 × 10^−6^
		AC005540.1	1.36 × 10^−3^
	Downregulated	AL365361.1	2.35 × 10^−7^
miRNA	Upregulated	hsa-miR-34a-5p	3.89 × 10^−16^
		hsa-miR-30d-5p	5.86 × 10^−14^
		hsa-miR-301a-3p	3.08 × 10^−13^
		hsa-miR-7-5p	8.75 × 10^−5^
	Downregulated	hsa-miR-101-3p	1.46 × 10^−17^
		hsa-miR-195-5p	5.78 × 10^−16^
mRNA	Upregulated	SAP30	4.37 × 10^−16^
		PGF	1.60 × 10^−14^
		PPFIA4	5.01 × 10^−13^
		KDELR3	3.10 × 10^−11^
		TKTL1	3.57 × 10^−10^
		SLC2A1	4.02 × 10^−9^
		ENO2	1.88 × 10^−5^

**Table 2:** A comparison between the abnormal expressions of eight prognostic model-related hypoxia mRNAs reported in this study and previous literature.

mRNA	Feature	Abstract	Citation
SLC2A1	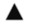	Through cell culture in vitro and immunohistochemical detection, Amann et al. showed that SLC2ZA1 was expressed at high levels and its overexpression promoted the growth and invasion of HCC. The knockdown of SLC2ZA1 inhibited glucose uptake and lactic acid secretion, and deactivated hypoxia transcription factor factor-1 (HIF1) under hypoxic conditions, thereby inhibiting the proliferation and metastasis of hepatocytes.	[18]
PGF	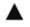	Alpini et al. showed by immunohistochemistry that PGF (PIGF) protein was notably higher in HCC tissues compared with normal tissues (*p* < 0.01). PIGF might recruit cyclic hematopoietic progenitor cells and macrophages into a growing tumor to induce tumor angiogenesis, which was associated with the early recurrence of HCC. Through quantitative real-time PCR (qRT-PCR) and immunohistochemistry, Vandewynckel et al. showed that inhibition of PGF deactivated pancreatic endoplasmic reticulum kinase, and further induced tumor angiogenesis to improve oxygen delivery, thus reducing tumor hypoxia in HCC.	[19,20]
TKTL1	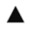	Zhang et al. showed via cell-based experiments that TKTL1 was overexpressed in human HEPG2 hepatoma cells. Silencing TKTL1 via siRNA, the total transketolase activity of HEPG2 cells was decreased and the proliferative activity was inhibited. Sun et al. showed that overexpressed TKTL1 participated in the occurrence and progression of Head and Neck squamous cell carcinoma by promoting aerobic glycolysis and accumulating HIF1α.	[21,22]
PFKP	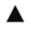	Through western blotting and immunohistochemical experiments, Sha et al. showed that PFKP were upregulated in HCC and its upregulation promoted cancer cell proliferation. Peng et al. showed that hypoxia (1% O_2_) induced oxidized ATM activation which increased the levels of PFKP and citrate synthase, promoted glucose to be converted into pyruvic acid and citrate, and enhanced the invasion and metastasis of hypoxic breast cancer cells.	[23,24]
KDELR3	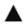	Bai et al. showed that overexpression of KDELR3 was correlated with poor prognosis by immunohistochemical experiments and real-time fluorescence quantitative PCR (RT-qPCR) analysis and KDELR3 could be a potential therapeutic target in hepatocytes and liver cancer tissues.	[25]
SAP30	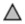 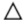	Snezhkina et al. showed that SAP30 was overexpressed in renal clear cell carcinoma (ccRCC) by qPCR analysis. The overexpression of SAP30 was positively associated with the expression of neuropilin and tolloid-like 2 (NETO2) gene and might activate NETO2 to cause tumor progression and poor prognosis of ccRCC. On the other hand, Chen et al. reported the upregulated expression of SAP30 in HCC via bioinformatics analysis and further constructed a glycolysis-related prognosis model including SAP30 which was connected to the poor prognosis of HCC patients.	[26,27]
ENO2	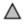	Zheng et al. showed by immunohistochemistry staining and western blotting that the expression of ENO2 was significantly higher in pancreatic ductal adenocarcinoma (PDAC) than in normal tissues.	[28]
PPFIA4	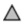	Huang et al. reported that the expression level of PPFIA4 was higher in Colon adenocarcinoma cells than in normal cells by qRT-PCR.	[29]

Note: 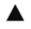 the mRNA experimentally reported to be upregulated in HCC in accord with our prediction in HCC. 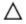 the mRNA computationally reported to be upregulated in HCC in accord with our prediction in HCC. 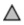 the mRNA experimentally reported to be upregulated in other cancers, and its expression in accord with our prediction in hepatocellular carcinoma.

**Table 3:** A comparison between the abnormal expressions of six prognostic miRNAs reported in this study and previous literature.

miRNA	Feature	Abstract	Citation
hsa-miR-301a-3p	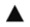	Hu et al. showed by western blot and Reverse Transcription-PCR (RT-PCR) that the expression of miR-301a-3p in HCC tissues was notably higher than that in normal tissues. The overexpression of miR-301a-3p downregulated the target gene VGLL4, and then enhanced the transcription activity of TEADs, promoting the proliferation, invasion, and chemoresistance of HCC cell.	[32]
hsa-miR-30d-5p	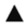	Zhuang et al. demonstrated that miR-30d-5p was upregulated in hepatocellular carcinoma HCCLM3 and MHCC97L cell lines compared with the normal cell line Huh7 (*p* < 0.005) by cell culture and transfection, qRT-PCR and Immunohistochemistry (IHC) staining. Silencing miR-30d-5p upregulated the target gene GLDC, decreased p62 expression and further induced cell autophagy, thus suppressing the progression and invasiveness of HCC cells.	[33]
hsa-miR-7-5p	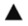	Through quantity PCR (qPCR) analysis, Zhang et al. showed that the expression of plasma hsa-miR-7-5p in HCC patients was significantly increased compared with normal and benign groups, and further constructed a prognostic model including the overexpression of miR-7-5p which was related to a poor diagnosis of HCC patients.	[34]
hsa-miR-195-5p	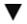	Xu et al. revealed via cell culture, tumor xenograft experiments and RT-qPCR analysis that miR-195-5p was expressed at low levels in HCC tissues, and upregulation of miR-195-5p directly downregulated PHF19 and thus inhibited the proliferation, invasion, and migration of HCC cells in vitro and the growth of xenograft HCC tumors in nude mice.	[35]
hsa-miR-101-3p	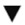	Yan et al. demonstrated by qRT-PCR and in vitro cell culture experiments that miR-101-3p was down-expressed in both HCC tissues and cell lines, and the linker LINC00052 could promote its expression and further down-regulate the miRNA target SOX9, thus inhibiting the proliferation and metastasis of HCC cells.	[36]
hsa-miR-34a-5p	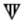 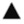	Ahmed et al. detected by RT-qPCR analysis that miR-34a-5p was significantly down-expressed in HCC than in normal liver tissues, and its downregulation increased the expression level of gene MCM2 to promote HCC development. However, Wan et al. revealed via HCC cell line culture that miR-34a-5p, an oxidative stress-responsive related miRNA, was upregulated in four HCC cell lines with oxidative stress models compared with normal cell lines.	[30,31]

Note: 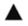 the miRNA experimentally reported to be upregulated in HCC in accord with our prediction in HCC. 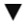 the miRNA experimentally reported to be downregulated in HCC in accord with our prediction in HCC. 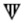 the miRNA experimentally reported to be down-regulated in HCC which was different from prediction in HCC.

**Table 4:** A comparison between the abnormal expressions of seventeen prognostic lncRNAs reported in this study and previous literature.

lncRNA	Feature	Abstract	Citation
BACE1-AS	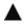	Liu et al. showed that BACE1-AS was overexpressed in HCC tissues by qRT-PCR analysis. Knocking out BACE1-AS upregulated the expression of MIR-377-3P, and deactivated epithelial–mesenchymal transition (EMT) process, inhibiting the invasion and migration of HCC cells.	[37]
CASC9	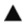	Yao et al. showed via qRT-PCR and western blot analysis that CASC9 was expressed at high levels in HCC tissues and positively associated with tumor node metastasis (TNM) stage, lymph node metastasis (LNM), tumor size, differentiation degree and α-fetoprotein (AFP). Downregulated CASC9 upregulated the expression level of miR-424-5p, enhanced apoptosis and decreased the proliferation, invasion and migration of HCC cells.	[38]
LINC00511	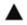	Through qRT-PCR analysis, Hu et al. showed that LINC00511 was up-regulated in HCC tissues and positively correlated with TNM stage, lymphatic metastasis, and poor prognosis. Knockdown of LINC00511 upregulated the level of miR-29c and attenuated the proliferation and migration of Huh7 and Hep3B cells.	[39]
LINC01139	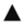	Li et al. showed via cell transfection and RT-PCR analysis that the expression of LINC01139 in HCC tissues was significantly increased than that in normal liver tissues. LINC01139 could downregulate miR-30 via acting as a sponge, and further upregulate MYBL2, accordingly aggravating the cancer progression and might serve as a prognostic biomarker for HCC patients.	[40]
MYLK-AS1	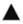	Through qRT-PCR and dual luciferase reporter analysis, Teng et al. detected that the overexpression of MYLK-AS1 was frequent in both HCC tumor tissues and cell lines. MYLK-AS1 overexpression directly downregulated miR-424-5p, upregulated E2F7 and further activated VEGFR-2 signaling, thus promoting the HCC progression.	[41]
RHPN1-AS1	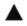	Zhang et al. showed that RHPN1-AS1 expression was elevated in both HCC tissues and cell lines by RT-PCR analysis and cell culture, respectively. Transcription factor STAT1-induced overexpression of RHPN1-AS1 stimulated CDCA5 expressions, further downregulated the expression level of the sponged miR-485 and then promoted the proliferation and metastasis of HCC cells.	[42]
SNHG1	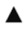	Li et al. determined via qRT-PCR and in vitro cell-based experiments that SNHG1 was overexpressed in both HCC tissues and cells. Knockdown of SNHG1 epigenetically increased CDKN2B and CDKN1A in the nucleus and repressed the expression of CDK4 by upregulating the sponged miR-140-5p in the cytoplasm, thus inhibiting cell cycle, growth, metastasis, and EMT process of HCC cells.	[43]
SNHG12	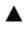	Through qRT-PCR analysis and in vitro cell-based experiments, Lan et al. detected that SNHG12 was expressed at higher levels in HCC. Abnormally high expression of SNHG12 downregulated the expression of miR-199a/b-5p, caused the upregulation of gene MLK3, and promoted the progression and metastasis of tumor cells by affecting the NF-κB signaling pathway.	[44]
LINC01703	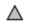	Wang et al. demonstrated via analyses of qRT-PCR and in vitro cell-based experiments that LINC01703 was over-expressed in NSCLC. The upregulation of LINC01703 expression downregulated the sponged miR-605-3p, and promoted the expression of MACC1, subsequently increasing the invasiveness of cancer cells.	[45]
AL365361.1	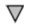 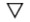	Cao et al. showed that AL365361.1 expression was downregulation in Ovarian cancer patient samples by RT-PCR analysis. Lv et al. showed in a data mining study that down-regulation of AL365361.1 and the other six abnormally expressed lncRNAs formed a lncRNA-based classifier which could efficiently predict the early recurrence after operating resection for HCC.	[46,47]
AP003469.4	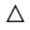	Fan et al. showed that AP003469.4 was expressed at high levels in HCC by data mining from TCGA and GEO. Up-regulation of AP003469.4 was a biomarker of diagnosis and dismal prognosis in HCC patients (Diagnostic ROC = 0.9048).	[48]
AC005540.1 AC012146.1 AC073529.1 AC090772.3 AC138150.2 AL390728.6	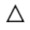	In this study, AC005540.1, AC012146.1, AC073529.1, AC090772.3, AC138150.2, and AL390728.6, which are calculated to be upregulated in HCC, were associated with a poor prognosis. The over-expression of these lncRNAs has not been reported in any cancer studies.	\

Note: 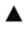 the lncRNA experimentally reported to be upregulated in HCC in accord with our prediction in HCC. 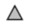 the lncRNA experimentally reported to be upregulated in other cancers in accord with our prediction in HCC. 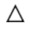 the lncRNAs upregulated in our prediction in HCC which have not been experimentally reported in cancers. 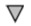 the lncRNA experimentally reported to be downregulated in other cancers in accord with our prediction in HCC. 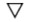 the lncRNA downregulated in our prediction in HCC which has not been experimentally verified in cancers.

## Data Availability

The data that support the findings of this study are openly available in TCGA database (http://cancergenome.nih.gov (accessed on 9 August 2021)).

## Supplementary Materials

The following supporting information can be downloaded at: https://www.mdpi.com/article/10.3390/ijms232113590/s1.
