# Supplementary material for: Identification of Hypoxia-Related Prognostic Signature and Competing Endogenous RNA Regulatory Axes in Hepatocellular Carcinoma
Source: Int J Mol Sci. 2022 Nov 5;23(21):13590. doi: 10.3390/ijms232113590 (PMC9658439; doi:10.3390/ijms232113590)
Supplement: Supplementary file 1 [file ijms-23-13590-s001.zip › ijms-1917710-Supplementary Document-S2-proofread.pdf]

Supplementary materials

Figure S1. lncRNA-miRNA-mRNA pairs with targeted regulation existing in ceRNA network

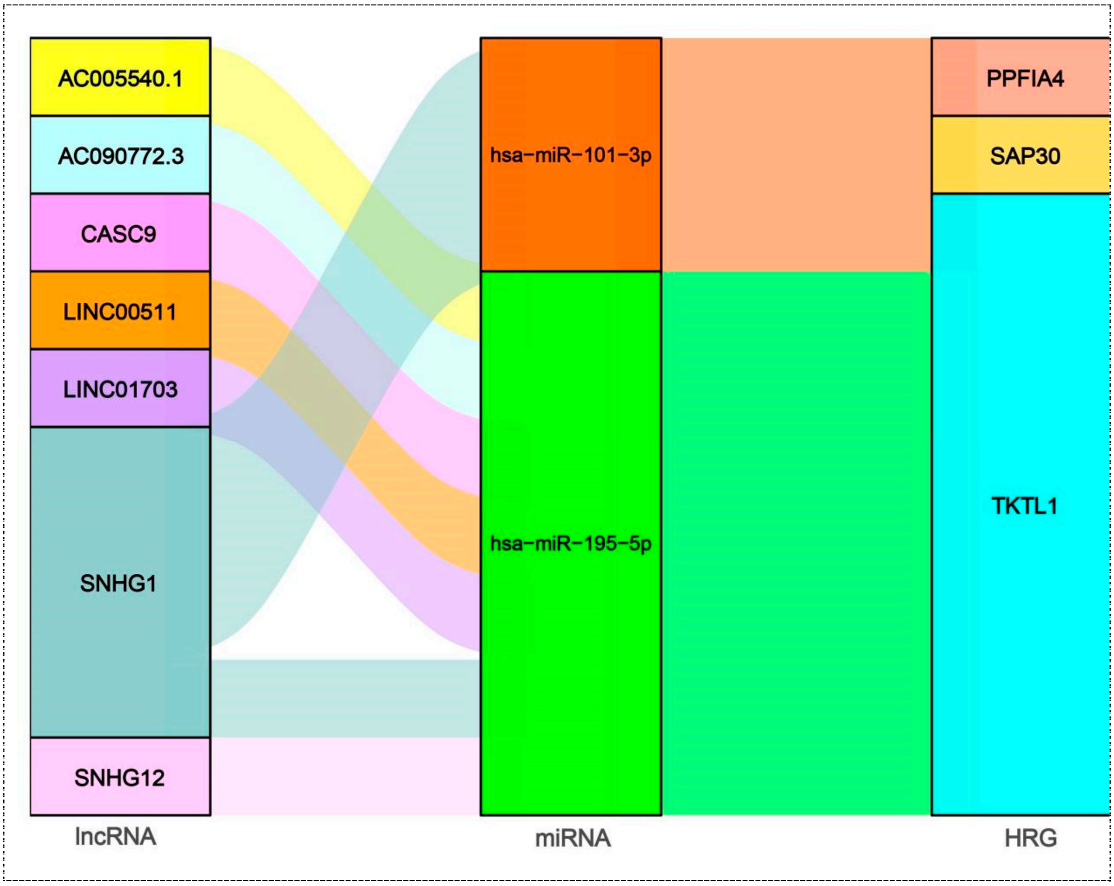

Table S4. Survival related DE-lncRNAs screened by univariate Cox analysis

id	HR	HR.95L	HR.95H	*P*-value
TMCC1-AS1	1.562172345	1.329864422	1.835061074	5.62E-08
DNAJC9-AS1	1.531626021	1.174656455	1.997076047	0.001638494
BACE1-AS	1.458556546	1.134663694	1.874905499	0.003218704
AL354824.2	1.445181061	1.246535614	1.675482254	1.06E-06
LINC01665	1.435707001	1.207630672	1.70685843	4.18E-05
AL592043.1	1.432953689	1.239211913	1.656985584	1.21E-06
KDM4A-AS1	1.43034944	1.173514187	1.743395643	0.000393398
AL355574.1	1.429475763	1.189335324	1.718103309	0.000140191
DDX11-AS1	1.412342023	1.19290224	1.67214875	6.14E-05
AC025524.2	1.409164231	1.2084477	1.643218675	1.21E-05
LINC01138	1.408310681	1.174723365	1.688345556	0.000215373
SNHG20	1.401585143	1.084084875	1.812072982	0.009995028
SREBF2-AS1	1.388937754	1.136593138	1.697307524	0.001320286
AL118511.1	1.387692464	1.171869401	1.643263637	0.000145363
SNHG3	1.386987607	1.18162849	1.628036763	6.30E-05
AC073611.1	1.373484703	1.132213903	1.666169463	0.001282613
AC012073.1	1.366678428	1.176886477	1.587077396	4.22E-05
GSEC	1.364683111	1.158411779	1.607683923	0.00020023
LINC01747	1.35218297	1.127379382	1.621813218	0.001144461
DEPDC1-AS1	1.346697063	1.149950455	1.57710532	0.00022088
AC012339.1	1.342266385	1.147109516	1.570625142	0.000240596
AC006252.1	1.341186417	1.148189158	1.566624272	0.00021292
AC091173.1	1.339261905	1.133665966	1.582143687	0.000591795
FAM242A	1.339211548	1.132994893	1.582961742	0.00061817
UBE2E1-AS1	1.338849772	1.091732739	1.641902497	0.005062866
AL158166.1	1.337268495	1.142905213	1.564685337	0.000286862
AL162574.2	1.337259803	1.135022418	1.575531684	0.000513025
AC084033.3	1.330737767	1.120581249	1.580307547	0.001121478
AP002852.1	1.328595353	1.145085897	1.541513713	0.000179416
AL513320.1	1.327888469	1.100768945	1.601869126	0.003045323
AP001469.3	1.327868133	1.139488748	1.547390249	0.000280392
AC007687.1	1.324919795	1.096371932	1.601110364	0.003587238
AC022639.1	1.323816241	1.131637182	1.548631899	0.000456215
SNHG1	1.32368043	1.06147552	1.650655005	0.01278691
AL442125.2	1.323388055	1.12258012	1.560116657	0.000846555
AL450468.2	1.321487993	1.133395986	1.54079469	0.000373122
AC069544.1	1.321128732	1.056532433	1.651990107	0.014596993
LINC02003	1.314218187	1.135340015	1.521279458	0.000251875
HDAC11-AS1	1.313869361	1.087418842	1.587477274	0.004679933
AP000526.1	1.313287328	1.126330693	1.531276397	0.000504614
GACAT3	1.311420557	1.109497667	1.550092378	0.00148311
AL024498.1	1.311365523	1.098108154	1.566038399	0.002758044

AC005332.5	1.309542455	1.071986676	1.599741377	0.008275293
AC025176.1	1.309144167	1.138882431	1.504859856	0.000151011
Z93403.1	1.306782245	1.142799474	1.494295259	9.19E-05
SNHG12	1.305306667	1.047996921	1.625792463	0.017385015
AC003092.1	1.304877918	1.078178161	1.579243989	0.006275219
AC004816.1	1.30292278	1.124169209	1.510099865	0.000440512
AC009005.1	1.302658184	1.146991905	1.47945102	4.66E-05
AC034229.4	1.302083291	1.109166055	1.528554618	0.001253941
MIR137HG	1.301065182	1.14057391	1.484139337	8.92E-05
AC114812.2	1.299549845	1.070321456	1.577871572	0.008136814
AC006372.1	1.298594929	1.120507172	1.504987055	0.000516858
LINC01060	1.298571089	1.162192592	1.45095304	3.93E-06
AC012676.1	1.298494305	1.120036134	1.505386665	0.00053462
AC110285.3	1.296857709	1.145800884	1.467829132	3.89E-05
AC010864.1	1.296432959	1.044637935	1.608919571	0.018456846
AC004923.4	1.2963718	1.090049679	1.541746103	0.003336981
LINC00462	1.295940199	1.138285148	1.475430827	8.96E-05
AC114803.1	1.295881238	1.13248806	1.482848467	0.000163698
AC005291.2	1.295788899	1.09974855	1.526775252	0.001961374
AC131009.1	1.295396949	1.106713941	1.516248412	0.001271333
AC011352.3	1.294980977	1.117420819	1.50075576	0.000591461
FILNC1	1.294910435	1.101815796	1.521845158	0.001708048
AC022007.1	1.293971911	1.074912548	1.55767398	0.006462803
AC026250.1	1.293689137	1.135277592	1.47420472	0.000111657
RHPN1-AS1	1.293423573	1.117210688	1.497429767	0.000574848
LINC00165	1.292622767	1.08528455	1.539571918	0.004008838
AL136529.1	1.29172709	1.103402557	1.512194134	0.001453431
C7orf71	1.291222943	1.064860881	1.565703763	0.00934945
AC068025.1	1.290965512	1.116561771	1.492610617	0.00056294
AC027237.2	1.290771454	1.063257941	1.566967791	0.009881021
LINC01134	1.289031806	1.108694093	1.498702851	0.000960474
SPRY4-AS1	1.287329197	1.137735938	1.456591469	6.14E-05
AL353708.1	1.286877488	1.061412353	1.560235912	0.010275407
AC010547.1	1.285815461	1.048209059	1.577282114	0.01588114
AC010789.1	1.283060513	1.129227104	1.457850485	0.000130737
AC089983.1	1.282461256	1.123438508	1.463993678	0.000230364
AL162431.1	1.281322452	1.066525395	1.539379403	0.008098498
U95743.1	1.279389623	1.125513335	1.454303345	0.000164274
AC097641.2	1.278882816	1.035659214	1.579227254	0.022282947
LINC01517	1.278304913	1.100927613	1.484260575	0.001275033
PICSAR	1.276644082	1.153172575	1.413335825	2.53E-06
AP003086.2	1.275815546	1.045369249	1.557062549	0.01655267
AC026356.2	1.275064821	1.092261432	1.488462608	0.00208599
AC025048.4	1.273061269	1.081486918	1.498571057	0.003714926

AC008622.2	1.273049193	1.131820152	1.43190086	5.72E-05
AL359397.1	1.271966232	1.074544977	1.505658794	0.005182989
AL359878.1	1.271493667	1.093193666	1.478874416	0.001834245
AJ239322.1	1.271265421	1.046459744	1.544364969	0.015633949
AC011773.4	1.2706617	1.077143016	1.4989478	0.004490452
LINC01460	1.270266209	1.088448268	1.48245561	0.002402855
AC097358.2	1.269908444	1.081954309	1.490513455	0.003457796
AC009902.3	1.26966149	1.067517379	1.510083424	0.00696735
AL136985.2	1.268922723	1.056426066	1.524162389	0.010866112
AF121898.1	1.26832724	1.057748982	1.520827736	0.010286418
AC096637.2	1.26670263	1.090104245	1.471910195	0.002027541
MYLK-AS1	1.266399865	1.07189759	1.49619575	0.005502278
LINC02008	1.264927569	1.114327733	1.435880763	0.000279387
LINC01954	1.263960737	1.132331673	1.410891157	2.98E-05
AC005540.1	1.2638928	1.076072372	1.484495887	0.004328359
CASC11	1.263719314	1.059410486	1.507429392	0.009284478
AC007405.1	1.263559625	1.075695097	1.484233711	0.004393537
AC011337.1	1.263424158	1.045354409	1.526985097	0.01557166
AC124861.1	1.262995294	1.067736834	1.493960928	0.006433077
AL133215.2	1.262945845	1.096483032	1.454680246	0.001206952
AL592466.1	1.26102633	1.101874459	1.443165682	0.000753521
MAGEA4-AS1	1.260932002	1.124251013	1.414230002	7.48E-05
AC002116.2	1.260027484	1.065127245	1.490591165	0.007020331
EGLN3-AS1	1.25987322	1.11660379	1.421525293	0.000176392
LINC02505	1.259702549	1.131712263	1.402167816	2.41E-05
AC069148.1	1.259382999	1.086281662	1.46006841	0.002235722
AP001011.1	1.259228424	1.061550539	1.49371713	0.008156768
AL591501.1	1.259203361	1.123220298	1.411649261	7.72E-05
AC106799.2	1.258233977	1.059580805	1.494131201	0.00879119
FOXD2-AS1	1.257948635	1.097415272	1.441965325	0.000986057
AL358214.1	1.257683577	1.066755524	1.48278396	0.006348596
DDN-AS1	1.257474393	1.0957064	1.443125503	0.001110803
LINC00412	1.255633524	1.022897933	1.541322448	0.029524082
AC022092.1	1.254152923	1.079899306	1.456524275	0.003006359
AC008892.1	1.253328865	1.077893675	1.457317433	0.003336562
AC013565.1	1.252407852	1.052412879	1.490408811	0.011230061
TDRKH-AS1	1.252186384	1.048518734	1.495415093	0.013025121
CYTOR	1.251904908	1.095864982	1.430163318	0.000940424
AC007998.3	1.251140002	1.097482992	1.426310307	0.000804335
AC090673.1	1.251077822	1.057981109	1.479417451	0.008821003
AL353807.2	1.250379193	1.039476449	1.504072678	0.017752062
AP002478.1	1.249397957	1.145434162	1.362797886	5.08E-07
AL160162.1	1.248598441	1.116423733	1.396421467	0.000100622
AL451074.2	1.247965047	1.051001896	1.481840104	0.011485807

AC010973.2	1.247942253	1.012649981	1.537905393	0.037719375
AC027088.3	1.247402274	1.045381099	1.488464288	0.014194763
AL355488.1	1.246331226	1.037502211	1.49719346	0.018600769
AL359694.2	1.245802847	1.040274697	1.491937406	0.016885225
AC006449.5	1.244798477	1.054400662	1.469577271	0.009726417
AC012103.1	1.244090233	1.076720777	1.437476215	0.003049467
AC007368.1	1.244009394	1.09767769	1.409848617	0.000627144
AL117335.1	1.243673493	1.110818128	1.392418542	0.00015477
AC145343.1	1.24336993	1.082231611	1.428500857	0.002098962
AL021154.1	1.243068807	1.075702512	1.436475271	0.003187855
AC116025.2	1.242903732	1.083441668	1.425835587	0.001909614
AC012640.1	1.242892468	1.060188518	1.457082077	0.007352304
AC019080.4	1.242755537	1.012853819	1.52484129	0.037311718
APCDD1L-DT	1.242392437	1.102080701	1.400568004	0.000385719
LINC02560	1.241781248	1.057995091	1.457493216	0.008053571
AL357079.1	1.240514727	1.032095299	1.491021991	0.021642232
MIR4435-2HG	1.239850685	1.057481129	1.453671068	0.008085724
AC012074.1	1.239358056	1.060373301	1.448554381	0.007005123
AP003062.1	1.239084226	1.1085491	1.384990272	0.000160426
AL513218.1	1.238608835	1.018370926	1.50647648	0.032177144
LINC01594	1.238179978	1.050088149	1.459962822	0.011043023
LRP4-AS1	1.236509593	1.066709368	1.433338845	0.004849966
AC114489.1	1.236403362	1.118388056	1.366871958	3.38E-05
AC244090.2	1.236151111	1.052504932	1.451840769	0.009777859
AC008667.1	1.235123344	1.018253746	1.498182237	0.032066063
MAFG-DT	1.235117494	1.105359241	1.380108084	0.000192413
AC090241.3	1.234577468	1.046777894	1.456069653	0.012315447
AC020658.4	1.234110655	1.045128473	1.457264965	0.013121208
LINC00501	1.233186763	1.099879663	1.382650889	0.000329433
DCST1-AS1	1.232884138	1.064194222	1.428313804	0.005291336
AC018814.1	1.230666377	1.044518242	1.449988779	0.013119868
AC099850.3	1.230423901	1.101997128	1.373817533	0.000227071
AC004221.1	1.23019736	1.062710895	1.4240802	0.005528375
AC074237.1	1.229941985	1.081604309	1.398623576	0.00159805
AC073352.1	1.229829431	1.039744348	1.454665689	0.015738169
AL590438.1	1.229570904	1.075186813	1.406122722	0.002536434
AC012313.8	1.229089634	1.047107683	1.442699116	0.011636126
FLJ36000	1.226630718	1.106948236	1.359253189	9.63E-05
AP002360.3	1.226369556	1.042348417	1.442878659	0.013894859
DLX2-DT	1.226326599	1.074465297	1.399651466	0.002488014
CU634019.6	1.225286956	1.040067041	1.443491685	0.015108135
LINC01775	1.224728442	1.032467552	1.452791184	0.019982042
PRR7-AS1	1.224346389	1.078292254	1.390183482	0.001790159
BX322234.2	1.22395067	1.081673948	1.384941595	0.001349783

AL355810.1	1.22340678	1.00348967	1.491519238	0.046106141
AC063948.1	1.223242965	1.012348947	1.478070734	0.03688051
LINC02393	1.223067494	1.04255498	1.434834732	0.013456081
AP000897.2	1.222136582	1.017378479	1.468104404	0.032023421
AL121757.1	1.221775188	1.067650745	1.398148802	0.00359771
AL121672.3	1.221504005	1.016539986	1.467794731	0.032759986
SNHG4	1.22139979	1.078766106	1.382892397	0.001596093
LINC01967	1.220048763	1.070208811	1.390867808	0.002931078
AC104823.1	1.22003075	1.01927795	1.460322996	0.03014876
SNAP47-AS1	1.219210765	1.004388164	1.479980493	0.045044346
AP001972.4	1.218811741	1.098749933	1.351992856	0.000184159
AC006557.1	1.218733423	1.02809306	1.444724427	0.022655862
LINC02293	1.217360196	1.073191696	1.380895746	0.002225759
WASIR2	1.217278177	1.067294389	1.388338753	0.003381689
AC087741.2	1.216461414	1.040726839	1.421870098	0.013838848
AC015722.2	1.216198148	1.078326368	1.371697828	0.001430774
AC138646.1	1.215817362	1.020752893	1.448158382	0.028511461
AC048344.4	1.215813449	1.02490614	1.442280697	0.024945515
AL450322.2	1.214582904	1.075513174	1.371635109	0.00172851
AC233280.1	1.213003148	1.04051121	1.414090135	0.013609765
AC011815.1	1.212764686	1.036624216	1.418834484	0.015987845
AL160286.2	1.211564066	1.035562719	1.41747811	0.016561399
AC004466.2	1.211370508	1.012294463	1.449596498	0.036316628
LINC02313	1.211321751	1.108656476	1.323494172	2.21E-05
AC023824.1	1.209558081	1.074450025	1.361655468	0.001642748
LINC01090	1.209454872	1.034051164	1.414611905	0.017368371
MYOSLID	1.209077577	1.024744469	1.426568896	0.024475128
AC011306.1	1.208221468	1.007284375	1.44924229	0.041532737
AC090192.2	1.208195991	1.115420028	1.308688671	3.49E-06
AC092171.2	1.207967304	1.039579158	1.403630495	0.013635148
AC091057.1	1.20790713	1.049558946	1.39014549	0.008422907
AL157714.2	1.207306148	1.04618984	1.393234841	0.009942136
AC026254.2	1.207267909	1.026876478	1.419348709	0.022539097
AC015961.2	1.207102495	1.045187207	1.394100908	0.010425194
AL161729.4	1.206948956	1.037306931	1.404334377	0.014936234
AC020915.2	1.206857221	1.029780467	1.414383354	0.020208958
AC048337.1	1.206678247	1.032460954	1.410292939	0.018200684
AC091057.4	1.205803361	1.044494721	1.392024025	0.010646679
AC022211.1	1.205120669	1.001202718	1.450571198	0.048532892
AC007128.2	1.205037557	1.048598711	1.384815277	0.008567976
AC131956.2	1.204598217	1.014770664	1.429935764	0.033374401
AC016642.1	1.203992099	1.033314665	1.402861125	0.017306454
LINC02046	1.20377694	1.065134771	1.360465324	0.002971224
AC010487.2	1.203590655	1.052148245	1.376831138	0.006915944

AC079174.2	1.202710574	1.008186006	1.434767707	0.040312274
AL445228.2	1.202612801	1.063742306	1.359612701	0.003208753
AC097461.1	1.20216885	1.020746148	1.415836784	0.027385803
AC092131.1	1.202105663	1.02529919	1.409401313	0.023343343
AC009902.2	1.202102091	1.021441993	1.414715126	0.026740235
TM4SF19-AS1	1.201655514	1.023556639	1.410743598	0.024804092
AL442067.1	1.201527688	1.000020188	1.443639639	0.049974811
AL049539.1	1.200997982	1.019187851	1.415240726	0.028749635
PCAT6	1.200471148	1.050702954	1.371587443	0.00720035
AC100812.1	1.200422455	1.011201695	1.425051081	0.036866085
AC012360.1	1.200033746	1.0138282	1.420438879	0.034039857
AC009121.2	1.199791481	1.046971028	1.374918274	0.008785938
AP003469.4	1.199589155	1.040452808	1.383065267	0.01220794
AC090772.3	1.199416415	1.0671599	1.348063901	0.002285437
LINC01649	1.199407811	1.030207242	1.396397771	0.01910209
AC026320.1	1.199086704	1.046087948	1.374462756	0.00913613
DSCR10	1.198542557	1.033813408	1.38951986	0.01635965
AC079089.1	1.198343601	1.041472439	1.378843388	0.011484017
AP001099.1	1.1975744	1.057171241	1.356624535	0.004599939
AC027228.2	1.197478687	1.06632153	1.344768126	0.002327398
AC025580.1	1.197472669	1.081741633	1.325585287	0.000510677
AL355987.4	1.197098546	1.063679848	1.347252118	0.002845795
AC083805.1	1.196997812	1.004911166	1.425801415	0.043919394
AP005205.2	1.196456166	1.051798107	1.361009634	0.006370579
AC138150.2	1.196346983	1.045004035	1.369608208	0.009380194
AC040174.1	1.196019197	1.082374215	1.32159645	0.000441626
UNC5B-AS1	1.195832398	1.041172148	1.373466555	0.011375399
PLBD1-AS1	1.195540855	1.08168322	1.321383109	0.000469358
AL035461.2	1.195395487	1.026854038	1.391600283	0.021351155
ERVMER61-1	1.195381611	1.097641281	1.301825306	4.12E-05
LINC01777	1.195255265	1.011148749	1.412883268	0.036629207
AL157395.1	1.195059599	1.014301325	1.408030741	0.033198653
AL606468.1	1.194291196	1.023916814	1.393014979	0.023763451
C10orf91	1.193912936	1.107892238	1.286612588	3.39E-06
LINC01096	1.193307303	1.054528088	1.3503503	0.005084336
AC009264.1	1.192539156	1.061291727	1.340017642	0.003077269
AC011921.1	1.192273869	1.008017182	1.410211061	0.040054026
AC025265.3	1.191983435	1.033328213	1.374998275	0.015959623
AC099786.1	1.191799985	1.01955335	1.393146524	0.027587505
LINC01508	1.191559778	1.101345916	1.289163273	1.28E-05
AC011447.3	1.190465098	1.054099975	1.344471287	0.004972836
AL121721.1	1.190086634	1.074953674	1.317550915	0.000801623
FIRRE	1.18915801	1.077827353	1.311988203	0.000551651
LINC01105	1.189150435	1.03528078	1.365889124	0.014269984

AC026355.1	1.188509185	1.053763846	1.340484481	0.004909037
AC078778.1	1.188467472	1.01754582	1.388099588	0.029292922
CEP83-DT	1.188213874	1.003540246	1.406871539	0.045395701
AL451069.3	1.188136039	1.083556718	1.302808817	0.000245371
C1orf147	1.188038697	1.01666162	1.388304542	0.030168764
AC100791.2	1.188002623	1.016539457	1.388387063	0.030293101
AC127496.1	1.187593333	1.003624409	1.405284599	0.045274671
AC002378.1	1.18752343	1.01382831	1.390977034	0.033156779
AC007128.1	1.187199633	1.079891325	1.305171118	0.000385098
AP005328.1	1.186802643	1.027578808	1.37069829	0.01980062
AL354760.1	1.186442462	1.006261343	1.398886805	0.0419279
AC245100.1	1.186119481	1.015781742	1.385021374	0.030933413
AC107959.3	1.186007064	1.075305193	1.30810561	0.000644386
FAM182B	1.185939193	1.05930368	1.327713475	0.0030773
AL353803.1	1.185489563	1.007674018	1.394682685	0.040153814
AC012146.1	1.185465319	1.031299766	1.362676564	0.016685981
LINC01424	1.185124212	1.000244162	1.404176551	0.049671137
AC024941.2	1.184752171	1.029752111	1.363083107	0.017798718
AP000695.2	1.183331925	1.05785638	1.323690504	0.003245871
AC026992.1	1.183294216	1.022440887	1.36945345	0.023966491
LINC01711	1.183230373	1.028796061	1.360847081	0.018383266
AC007064.2	1.182762232	1.017799631	1.374461589	0.028511594
AL390728.6	1.18274103	1.002500417	1.395387294	0.04663857
AL591845.1	1.182735164	1.031425507	1.35624188	0.016261634
AC090921.1	1.181758114	1.050351161	1.32960508	0.005490444
AL450322.1	1.181563107	1.001060412	1.394612513	0.048553198
AC090578.1	1.181015449	1.084065811	1.286635439	0.00014069
LINC00632	1.180776685	1.084563009	1.285525661	0.000127166
AC099786.2	1.180697284	1.03646135	1.345005366	0.012465651
LINC00336	1.180473231	1.007557633	1.383064356	0.04005949
AC007773.1	1.180400356	1.033355465	1.348369509	0.014552223
AP003469.2	1.17987506	1.079028827	1.2901464	0.000285089
LINC02434	1.179608292	1.044573336	1.332099599	0.00774469
AC011287.1	1.179373862	1.041527077	1.335464758	0.009280224
AL928654.1	1.179257684	1.001350109	1.38877369	0.048139602
LINC01293	1.179110758	1.018141468	1.36552947	0.027804451
BX119904.2	1.179057605	1.037118028	1.340422977	0.011840736
LINC01269	1.179025942	1.066158957	1.303841386	0.001337773
AC016405.3	1.179020574	1.042963788	1.332826249	0.008479163
AC073529.1	1.178930395	1.005133022	1.38277904	0.043085213
LINC01230	1.178768818	1.02945314	1.349741793	0.017311787
AC100872.1	1.17757222	1.05508933	1.314273867	0.00353486
AP005264.3	1.177363662	1.01143676	1.370510987	0.03514453
ELDR	1.176306173	1.007185066	1.373825188	0.040328767

AL355802.2	1.17561624	1.006327167	1.373383914	0.04140318
AC093730.1	1.17554004	1.009880998	1.368373491	0.036902901
LINC02561	1.174314714	1.057100888	1.304525485	0.002744595
AC015574.1	1.174118771	1.003486395	1.373765399	0.045134208
FER1L6-AS2	1.173547926	1.052295603	1.308771728	0.00402671
LINC00862	1.173367999	1.04509221	1.317388502	0.006796892
AC079160.1	1.172916014	1.034545558	1.329793515	0.012766008
SAMMSON	1.172382953	1.029782473	1.334730221	0.016239763
SIRLNT	1.172056047	1.000923118	1.372448446	0.048675665
LINC00200	1.171658421	1.076209686	1.275572477	0.00025819
AL162431.2	1.171348851	1.002812568	1.368209948	0.045999339
MELTF-AS1	1.171343449	1.035065061	1.325564476	0.012207443
LINC01477	1.17089476	1.007313187	1.36104099	0.039891682
LINC01136	1.170678244	1.051428217	1.303453273	0.004042028
AC018558.1	1.170639336	1.009514197	1.357481111	0.037042399
LINC00520	1.170548789	1.011340771	1.354819767	0.034759622
AC106799.3	1.170509719	1.002327181	1.366911951	0.04666423
AC093515.1	1.169919996	1.060772272	1.290298429	0.001685841
AL138720.1	1.169855741	1.013752821	1.349996199	0.03180228
PRRT3-AS1	1.169530326	1.045030975	1.308861859	0.006392109
AF254983.1	1.168873746	1.029372496	1.327280299	0.016110047
AL139289.2	1.168806352	1.002922834	1.362127016	0.045788718
AC109439.2	1.168246313	1.038938986	1.313647352	0.009370665
AL161431.1	1.167896908	1.064489299	1.281349834	0.001033776
DUXAP8	1.167551951	1.064647777	1.28040239	0.000999407
VLDLR-AS1	1.167445143	1.05444374	1.29255655	0.002876926
AC112236.1	1.167183989	1.032473941	1.319470071	0.01348445
AC132872.2	1.166973546	1.000764998	1.360786257	0.048870623
AL359853.1	1.166929296	1.036555902	1.313700478	0.010651135
AC073370.1	1.166187574	1.010032421	1.346484953	0.036076634
AL022324.3	1.165207939	1.013518949	1.339599564	0.031660513
AC006058.3	1.164103531	1.004442025	1.3491441	0.04350273
AC108752.1	1.164024852	1.070737035	1.265440357	0.00036587
LINC01029	1.163889199	1.02308319	1.324074211	0.021063934
PLUT	1.162834951	1.051152437	1.286383474	0.00340817
CASC16	1.16252122	1.007837043	1.340946532	0.038722707
AL359636.1	1.162435887	1.005929518	1.343292118	0.04134092
AL121832.3	1.16185487	1.005349183	1.342724261	0.042129948
LINC02154	1.161759844	1.058807375	1.274722832	0.00154053
LINC02577	1.161154901	1.02015578	1.321641979	0.023692519
AC121247.1	1.160967781	1.024395321	1.315748092	0.01941699
LINC00622	1.160493588	1.017088688	1.32411793	0.026984257
LINC01116	1.159928289	1.058169506	1.271472698	0.001540769
TGFB2-AS1	1.159535018	1.041830936	1.290537083	0.006721635

LINC00628	1.15950091	1.016850794	1.322162867	0.027143659
AP000844.2	1.158594288	1.054373389	1.273117037	0.002206852
AC145423.2	1.158527967	1.005140429	1.335322916	0.042283212
MINCR	1.158026575	1.004848948	1.334554363	0.042684243
AC078906.1	1.157221174	1.003710186	1.334210677	0.044329155
CDKN2B-AS1	1.156619755	1.024567305	1.305691927	0.018655385
LINC01731	1.156503778	1.021162355	1.309782899	0.022036035
AC009646.2	1.156043178	1.014791306	1.316956326	0.029198931
AL606489.1	1.155969505	1.017136625	1.313752216	0.026402031
AP000525.1	1.155132244	1.032843802	1.291899607	0.011537139
AC040174.2	1.154934319	1.021997355	1.305163144	0.020959492
AC105219.3	1.153914107	1.020563808	1.304688404	0.022322558
LINC02345	1.153596869	1.024993463	1.298335827	0.017821297
AC040970.1	1.152730174	1.052551929	1.262443038	0.002183135
AC245595.1	1.152293504	1.031414861	1.28733875	0.012175889
AP001453.2	1.151283909	1.014429238	1.306601377	0.029121389
AC122710.1	1.150766575	1.05973111	1.249622379	0.000838712
AC016708.1	1.150731012	1.016628952	1.302522282	0.026361673
LINC00346	1.150491255	1.035917083	1.277737524	0.008812067
AC017076.1	1.150202067	1.019910823	1.297137715	0.022526141
AP003900.1	1.150185745	1.033916691	1.27952983	0.010070577
AP003419.3	1.149758929	1.0093928	1.309644367	0.035667761
AL365295.1	1.149249488	1.016548146	1.299273813	0.02627369
LINC02487	1.148334753	1.031566453	1.278320656	0.011471526
AL355388.2	1.148274071	1.017450656	1.295918711	0.025072393
FP325330.3	1.148198343	1.001687828	1.316138021	0.047236087
AC138904.1	1.147848656	1.022611276	1.288423634	0.01932037
AC116337.3	1.147652336	1.002090178	1.314358641	0.04657583
AC010980.2	1.147489737	1.028604196	1.280116007	0.013687856
RNF144A-AS1	1.146974312	1.053031954	1.249297392	0.001660042
AC092115.3	1.146431258	1.042962251	1.260165099	0.004631762
SRGAP3-AS2	1.146400085	1.006358609	1.305929262	0.039848532
LINC01353	1.146278903	1.005384374	1.306918386	0.041329018
AL359313.1	1.145933073	1.056160736	1.243335946	0.001065175
LINC00578	1.14519552	1.010544084	1.297788785	0.033643986
AC016877.3	1.144806599	1.02538115	1.278141449	0.0161343
ATP2A1-AS1	1.144629683	1.016101484	1.289415606	0.026228666
LINC00460	1.144106425	1.018934042	1.284655785	0.022771225
AC005993.1	1.144050265	1.001362321	1.307070359	0.04770419
AC108868.2	1.143551652	1.004697735	1.301595829	0.042262781
AL163952.1	1.143407585	1.044609377	1.251550039	0.003654921
LINC01297	1.143294756	1.030390293	1.268570665	0.011593212
LINC00958	1.142630982	1.063565178	1.227574565	0.00026801
LINC01804	1.141420244	1.011575457	1.287931774	0.031813134

AC233266.2	1.140611021	1.021509403	1.273599144	0.019377934
LINC00513	1.13998447	1.022768425	1.270634252	0.01795044
AC006946.2	1.139561729	1.034793676	1.254937061	0.007929777
AC099066.2	1.139357465	1.031751698	1.258185894	0.009951476
MSC-AS1	1.139238822	1.048867627	1.237396464	0.001992166
PINCR	1.139160678	1.007956303	1.287443758	0.036897478
AL512598.1	1.13903889	1.016865866	1.275890592	0.024519838
AP005230.1	1.138117182	1.02456194	1.264258089	0.015846564
AC131391.1	1.137211824	1.017817612	1.27061147	0.02308446
LINC01694	1.13661947	1.053475876	1.226325015	0.000952852
HCG20	1.136187258	1.0131212	1.274202421	0.029048363
LINC01703	1.136136444	1.015869652	1.270641384	0.025366704
CASC15	1.135699647	1.034880408	1.246340812	0.007300381
LINC01992	1.134604396	1.018761428	1.263619824	0.02154818
LINC00839	1.13430044	1.024760408	1.255549569	0.01501581
LINC02525	1.133553562	1.04000861	1.235512538	0.004335358
AL356215.1	1.133316649	1.012657954	1.268351887	0.029333935
AC023347.1	1.133219481	1.018740123	1.260563282	0.021353936
LINC01518	1.1331444	1.028768839	1.248109568	0.011237355
AL589182.1	1.132860552	1.009325708	1.27151525	0.034214715
AC074135.1	1.132250933	1.029573063	1.245168722	0.010441853
CU104787.1	1.132086478	1.007962581	1.271495409	0.036276256
LINC01224	1.132036151	1.056742301	1.212694757	0.000413029
AL133467.2	1.131981584	1.028300737	1.246116297	0.011426843
AL138974.1	1.131740359	1.018917878	1.25705542	0.020902489
AL358394.1	1.131070864	1.011940096	1.264226316	0.030083513
AC015912.3	1.130458814	1.000164709	1.277726678	0.049692677
AC097652.1	1.130270331	1.013018007	1.261094089	0.028421111
MIR663AHG	1.129567909	1.052796831	1.211937217	0.000692157
DSCR4-IT1	1.129523726	1.017677072	1.253662761	0.022060385
AC006273.1	1.129381949	1.019142668	1.251545664	0.020243385
AC092667.1	1.129225342	1.017390383	1.253353573	0.022372929
LINC02152	1.12910127	1.004785418	1.268797949	0.041332536
LINC01511	1.128384176	1.014788185	1.254696171	0.025672809
AC009275.1	1.128346884	1.041267027	1.22270912	0.003210878
AC109830.1	1.127221259	1.025990152	1.238440509	0.012617023
LINC02588	1.126750526	1.0433597	1.216806389	0.002350846
LINC00858	1.126684129	1.0151381	1.250487126	0.024933896
ZFPM2-AS1	1.126436809	1.058046765	1.19924745	0.000194855
HOXA-AS3	1.12582234	1.010378908	1.254456058	0.031791566
AC124067.4	1.125638947	1.053918778	1.202239743	0.000426078
AC079209.1	1.125565295	1.030178527	1.22978416	0.008843912
AL133370.1	1.124801175	1.026679716	1.232300262	0.011558469
AC016717.2	1.12425191	1.047232809	1.206935407	0.001218237

LINC00601	1.12397237	1.00982801	1.251018862	0.032438926
AL139412.1	1.123811919	1.00377597	1.258202296	0.042830545
AL139042.1	1.123775837	1.013726165	1.245772454	0.02647142
AC064853.1	1.123384626	1.0068669	1.253386141	0.037301111
ZIM2-AS1	1.12328715	1.018685251	1.238629911	0.019744193
AL133467.4	1.122973362	1.024507432	1.230902903	0.01324629
AL137026.2	1.121594084	1.014352904	1.240173201	0.025228478
LINC01532	1.119489352	1.026678022	1.220690793	0.010581581
CASC8	1.11890762	1.010720249	1.238675354	0.030350479
AC103770.1	1.118754664	1.002136743	1.248943326	0.045720203
LINC02466	1.116967639	1.010651123	1.234468233	0.030191342
LINC00668	1.116810601	1.045267816	1.193250094	0.001072929
LINC01748	1.11634021	1.04034274	1.197889326	0.002217727
LINC01793	1.116047577	1.027797474	1.211875127	0.008992843
TMEM132D-AS1	1.116003207	1.044488415	1.192414527	0.0011616
IGFL2-AS1	1.115887946	1.007525703	1.235904855	0.035394521
AC132807.2	1.115606392	1.016940381	1.223845219	0.020584438
ADGRD1-AS1	1.113325462	1.026400511	1.20761201	0.009647429
AL035045.1	1.112725976	1.00653594	1.230119113	0.036863766
SLC7A11-AS1	1.112321088	1.007246327	1.228357125	0.03550274
LINC00491	1.111253013	1.023552585	1.206467823	0.011903983
AC091987.1	1.1108447	1.016368062	1.214103429	0.020451156
LINC00664	1.109311306	1.008291034	1.220452759	0.033216892
AC011632.1	1.109029345	1.036348989	1.186806859	0.00276811
USP2-AS1	1.108790392	1.010291144	1.216892912	0.029580093
AC109588.1	1.108463523	1.033870046	1.188438901	0.003766586
LINC01559	1.107825016	1.037419384	1.183008806	0.002239307
AL136537.2	1.107610966	1.030263083	1.190765808	0.0056545
LUCAT1	1.106466703	1.035990305	1.181737473	0.002587376
AC005381.1	1.105428757	1.014439831	1.204578823	0.022190494
LINC00879	1.103777618	1.015996585	1.19914284	0.01952708
MIR646HG	1.103518047	1.014677246	1.200137369	0.021436303
GPR1-AS	1.103368878	1.02108155	1.19228761	0.01286327
AL590004.3	1.102248143	1.016964442	1.194683825	0.017817541
LINC02475	1.101650821	1.020580053	1.189161526	0.01305371
AL451069.1	1.101153063	1.001284218	1.210982901	0.046986574
LINC01929	1.101020588	1.002710154	1.20896984	0.043729019
LINC02327	1.100843604	1.011139205	1.198506233	0.026732713
AC114485.1	1.09993191	1.009320372	1.198678081	0.029896023
HOXC-AS1	1.097299892	1.000821791	1.203078374	0.047989851
AC010615.2	1.096850476	1.007123114	1.194571895	0.033756554
AC093895.1	1.095545588	1.018479402	1.178443211	0.014207331
FAM230C	1.095301688	1.014857003	1.182122983	0.019341318
AL117329.1	1.094001691	1.003115869	1.193122089	0.042329372

LINC02562	1.093675014	1.002547708	1.193085403	0.043665555
AC069277.1	1.09228371	1.007611798	1.184070796	0.032020061
TCL6	1.091779829	1.01020916	1.179937029	0.026668166
BBOX1-AS1	1.091395189	1.008640634	1.180939393	0.029719302
LINC02253	1.091329529	1.01115773	1.17785792	0.024768822
AC006305.1	1.090992491	1.0105928	1.177788536	0.025764046
AC023090.1	1.090295775	1.009908493	1.177081771	0.026947321
LINC00942	1.090152634	1.035619934	1.147556866	0.000978197
LINC01139	1.088979739	1.028021214	1.153552919	0.003728735
AC016710.1	1.088349134	1.008573028	1.17443537	0.029276107
AC116049.2	1.088282955	1.001274687	1.182852023	0.046598961
LINC02159	1.087671851	1.000547657	1.182382516	0.048516457
AL139023.1	1.087551133	1.001350849	1.181171882	0.046370606
G2E3-AS1	1.085999045	1.00425008	1.174402621	0.038811529
AL109615.3	1.085815439	1.010510848	1.166731827	0.024762479
AC007639.1	1.085588488	1.005132809	1.172484228	0.036592975
LINC01436	1.085313238	1.019074171	1.155857796	0.01083347
AC006206.2	1.084174144	1.0067977	1.167497278	0.032411991
LINC01234	1.082849796	1.026037665	1.142807639	0.003794046
AC068506.1	1.082299607	1.000423412	1.170876677	0.048779808
LINC02188	1.082069522	1.000233813	1.170600748	0.049322809
LHFPL3-AS2	1.081550544	1.01505469	1.152402517	0.015456061
CASC20	1.079030761	1.0036287	1.160097737	0.039593558
AC005186.1	1.078949281	1.005868865	1.157339283	0.033712711
LINC00511	1.078339002	1.005196693	1.156803451	0.035326243
LINC00648	1.076323798	1.011691962	1.145084632	0.019919433
LINC01194	1.07567411	1.010848606	1.144656859	0.021436715
PART1	1.075572956	1.013114545	1.141881922	0.016994328
CASC9	1.073287297	1.023044183	1.125997919	0.003836081
AF279873.3	1.073221837	1.004684453	1.146434691	0.035837341
ELFN1-AS1	1.072396724	1.002414877	1.147264231	0.042354961
DSCR4	1.072216911	1.003510081	1.145627857	0.039049426
AC010894.3	1.071959647	1.001796384	1.147036967	0.04422744
LINC02377	1.070388014	1.000566018	1.145082364	0.048108881
LINC01667	1.067105794	1.011457644	1.125815581	0.017459885
LINC01446	1.066237313	1.002388526	1.134153053	0.041781912
LINC01833	1.064999189	1.001398484	1.132639294	0.045022279
LINC02055	1.063679628	1.000694997	1.130628568	0.047448757
AC245100.6	1.062831566	1.001796351	1.127585399	0.043441333
LINC02506	1.054800682	1.001867533	1.110530527	0.04225594
AC079466.1	1.054708499	1.010684003	1.100650662	0.014345773
LINC00221	1.048417732	1.002667753	1.096255203	0.037801898
DIO3OS	0.94054187	0.885999888	0.998441446	0.0443109
LINC01018	0.940053147	0.901238729	0.980539218	0.004060093

FAM99A	0.937176058	0.892461588	0.98413083	0.009287674
AL035661.1	0.933557741	0.876926144	0.993846587	0.031296675
FAM99B	0.925029179	0.869509594	0.984093778	0.013598766
LINC01093	0.923278773	0.866394511	0.983897845	0.013882277
AC092384.2	0.922231502	0.852701903	0.997430568	0.042940061
TSPEAR-AS1	0.921571036	0.857912901	0.98995268	0.025320605
AL161645.1	0.91903781	0.853067499	0.990109805	0.026317594
AC136475.3	0.917210023	0.851238311	0.9882946	0.023260426
AP003716.1	0.916997124	0.858620911	0.979342239	0.009824272
AC079360.1	0.915257935	0.848348	0.987445114	0.022245107
AC004160.1	0.914947302	0.847797948	0.987415183	0.022276912
AL137798.1	0.913762878	0.840765928	0.993097567	0.033752439
AC116025.1	0.908920584	0.82701389	0.998939241	0.047480243
AC020978.4	0.906526993	0.828833676	0.991503137	0.031823477
AL391095.2	0.900094128	0.825615939	0.981290938	0.016915204
AC253536.6	0.898531386	0.820407528	0.984094641	0.02114203
AL354872.2	0.898102977	0.823190639	0.979832519	0.015587357
AC138356.1	0.897287231	0.809325109	0.994809583	0.039511465
AC254629.1	0.896944212	0.811468939	0.991422937	0.033291703
AL590079.1	0.895659524	0.807307885	0.993680351	0.037561563
AC079061.1	0.892036414	0.825065222	0.964443711	0.004115569
AC243836.1	0.890961567	0.796403061	0.996747191	0.043708002
AC016395.1	0.888379585	0.803005431	0.982830571	0.02168074
AC004832.5	0.888371166	0.794583645	0.99322876	0.037588107
AC099508.2	0.88802402	0.815203486	0.96734947	0.006520714
AC115619.1	0.88465895	0.822533825	0.951476322	0.000970733
AL161668.4	0.884316818	0.81008829	0.965346918	0.005988768
AC004160.2	0.883684123	0.792456595	0.985413755	0.026130426
AL161740.1	0.883491291	0.788720519	0.989649493	0.032380586
SERTAD4-AS1	0.883035184	0.788492181	0.988914228	0.031326206
SLC6A1-AS1	0.879459671	0.792315365	0.976188709	0.015838307
AL078590.3	0.878969594	0.783561247	0.985995097	0.027768181
HAO2-IT1	0.876787617	0.776275356	0.990314221	0.034290529
LINC02127	0.876532566	0.774479626	0.992032991	0.036922654
AL139385.1	0.876135944	0.794992835	0.965561144	0.007659479
AC118754.1	0.875796269	0.777377447	0.986675273	0.029219179
AL365361.1	0.87437808	0.793996039	0.962897785	0.006364501
MIR99AHG	0.873862815	0.787877059	0.969232714	0.010732621
CLRN1-AS1	0.872071637	0.766939625	0.991615135	0.036758975
AC106822.1	0.871544778	0.781543249	0.971910768	0.013425052
LINC02362	0.870549282	0.80393635	0.94268166	0.000641862
AL133467.1	0.86812278	0.775086627	0.972326362	0.014477744
SNX29P2	0.868031199	0.758771444	0.993023879	0.039211801
AC019257.1	0.862697601	0.779049643	0.95532699	0.004536501

AC009159.3	0.859034008	0.740260628	0.996864347	0.045356909
AL354714.1	0.857781242	0.740085859	0.994193646	0.041620029
AC010280.2	0.857311685	0.779706793	0.942640659	0.001472005
LINC00702	0.855916957	0.761378898	0.962193514	0.009178277
F11-AS1	0.854735806	0.783981243	0.931875992	0.000370341
AC139887.4	0.854039457	0.739610932	0.986171731	0.031580091
AC011365.1	0.849390448	0.756187031	0.954081602	0.005912106
AL135999.1	0.849329426	0.735472404	0.980812428	0.026163108
AL031722.1	0.846850861	0.738256025	0.971419612	0.017592099
CPS1-IT1	0.845660802	0.770779334	0.92781703	0.00039449
AC007364.1	0.843262007	0.731147732	0.972567897	0.019175286
VIPR1-AS1	0.839303503	0.71690187	0.982603616	0.029393483
LINC02259	0.83500129	0.725024434	0.961660216	0.01233152
AC025259.3	0.833846589	0.730180969	0.952229876	0.007304756
AC007278.1	0.833076244	0.705968483	0.983069421	0.030609119
AC092378.1	0.832678686	0.706852624	0.980902907	0.02847742
AL109933.1	0.830725101	0.718361404	0.960664354	0.012377807
AP001065.1	0.826788424	0.76059539	0.898742099	7.92E-06
AC016586.1	0.826672562	0.701633261	0.973995338	0.022914198
LINC00402	0.825486403	0.745236697	0.91437768	0.000237475
B4GALT1-AS1	0.822715372	0.70495147	0.960152026	0.013290851
AC012629.2	0.821364557	0.684894662	0.985027001	0.03377946
AP001107.5	0.821100695	0.699173421	0.964290592	0.016246622
LINC02427	0.818690922	0.69751008	0.960924932	0.014379145
SOCS2-AS1	0.818576525	0.734412745	0.912385483	0.000298748
TMEM220-AS1	0.813961751	0.737985355	0.897760001	3.83E-05
AC009084.1	0.813740919	0.670071441	0.988214454	0.03756402
LINC01537	0.813256997	0.709730427	0.931884724	0.002925774
PGM5P3-AS1	0.807730949	0.668331268	0.976206436	0.027167428
AL136084.3	0.802847497	0.700833973	0.919710129	0.001539661
LINC02580	0.799724669	0.702366439	0.91057817	0.000739984
AL359076.1	0.798827644	0.658762941	0.96867259	0.022397521
AC022784.5	0.7978765	0.704971855	0.903024575	0.000350312
C9orf163	0.796419309	0.680474255	0.932120079	0.004573599
AL139246.1	0.792138578	0.674353982	0.930495769	0.004553509
AC090618.1	0.786058208	0.663246388	0.931610811	0.005482094
AC015908.3	0.779866384	0.705544989	0.862016718	1.14E-06
AC107396.1	0.776277192	0.607740784	0.991551488	0.042570029
AL138995.1	0.776003685	0.649757149	0.926779674	0.005121067
CLLU1	0.757385225	0.57363355	0.999997959	0.049998317
AC133919.1	0.729244077	0.59447432	0.894566688	0.002456278
HTR2A-AS1	0.692235701	0.555679731	0.862349728	0.001034688

Table S5. Survival related DE-miRNAs screened by univariate Cox analysis

id	HR	HR.95L	HR.95H	*P*-value
hsa-miR-3680-3p	1.45243273	1.191463351	1.770562925	0.000221141
hsa-miR-7-5p	1.416476056	1.21678896	1.648933778	7.10E-06
hsa-miR-5003-3p	1.409974013	1.167088006	1.703407718	0.000368357
hsa-miR-3682-3p	1.405401998	1.170892561	1.6868796	0.000258417
hsa-miR-3677-3p	1.394604984	1.191047586	1.632951602	3.60E-05
hsa-miR-3127-5p	1.353322907	1.099885924	1.665157132	0.004238155
hsa-miR-760	1.32322982	1.12652023	1.554288249	0.000647776
hsa-miR-548f-3p	1.283794308	1.082110801	1.523067531	0.004170762
hsa-miR-5010-3p	1.274203531	1.052735354	1.542262861	0.012863673
hsa-miR-1180-3p	1.268723141	1.089559993	1.477347204	0.002182137
hsa-miR-3189-3p	1.259054822	1.091395514	1.452469819	0.00158062
hsa-miR-653-3p	1.242907299	1.069710191	1.444146804	0.004509981
hsa-miR-421	1.242767495	1.04448596	1.478690097	0.014255721
hsa-miR-3660	1.238887284	1.071993317	1.431764246	0.003712011
hsa-miR-301a-3p	1.238306098	1.046744514	1.464924796	0.0126756
hsa-miR-326	1.232744074	1.06886406	1.421750445	0.004040251
hsa-miR-3677-5p	1.231964511	1.053817479	1.440227161	0.008851204
hsa-miR-4746-5p	1.227594666	1.046388389	1.440180987	0.011855162
hsa-miR-561-5p	1.226239599	1.084079844	1.387041336	0.001178264
hsa-miR-4784	1.225636432	1.031706258	1.456019726	0.020605092
hsa-miR-188-5p	1.22326964	1.024068468	1.461219303	0.026268376
hsa-miR-147b-3p	1.209614641	1.043854615	1.401696711	0.011382174
hsa-miR-877-5p	1.193353601	1.012937834	1.40590347	0.0345431
hsa-miR-1266-5p	1.190747307	1.033441021	1.37199813	0.015735698
hsa-miR-4664-3p	1.185623636	1.00997252	1.39182342	0.03741031
hsa-miR-137-3p	1.182416114	1.069974758	1.30667369	0.001014106
hsa-miR-9-3p	1.179752853	1.081650674	1.286752577	0.000190036
hsa-miR-4661-5p	1.168696887	1.025668094	1.331670959	0.019259288
hsa-miR-200c-5p	1.163075311	1.005800396	1.344942977	0.041551076
hsa-miR-31-5p	1.15510321	1.021721631	1.305897209	0.021266425
hsa-miR-9-5p	1.14953132	1.067537945	1.237822282	0.000223398
hsa-miR-3200-3p	1.138135351	1.020534361	1.269288058	0.020059047
hsa-miR-767-3p	1.130219138	1.026209941	1.244769953	0.012946734
hsa-miR-1270	1.119266195	1.003101106	1.248883893	0.043868353
hsa-miR-4652-5p	1.085228336	1.013811001	1.161676624	0.018528422
hsa-miR-767-5p	1.083948302	1.032023203	1.138485954	0.001288632
hsa-miR-105-5p	1.079724981	1.030432352	1.131375613	0.001293639
hsa-miR-5589-5p	0.921520703	0.852421466	0.996221283	0.039863301
hsa-miR-5589-3p	0.916956171	0.844548695	0.995571509	0.038855206
hsa-miR-122-3p	0.909269739	0.842486983	0.981346269	0.014534962
hsa-miR-2114-5p	0.89891145	0.811867814	0.995287386	0.040279722
hsa-miR-204-5p	0.881521848	0.791193717	0.982162462	0.022238169

hsa-miR-490-3p	0.877296474	0.772681467	0.996075532	0.043315068
hsa-miR-195-5p	0.848261876	0.73960156	0.97288628	0.01862295
hsa-let-7c-3p	0.834079742	0.732505935	0.949738403	0.006175776
hsa-let-7c-5p	0.809452846	0.714222941	0.917380096	0.000931914
hsa-miR-542-5p	0.794126886	0.673958183	0.935722018	0.005894615
hsa-miR-30d-5p	0.789898158	0.658764716	0.947134971	0.0108853
hsa-miR-34a-5p	0.776302645	0.651006598	0.925713808	0.004810526
hsa-miR-139-3p	0.775900481	0.682837306	0.881647138	9.93E-05
hsa-miR-660-5p	0.775482851	0.631550108	0.952218429	0.015208942
hsa-miR-139-5p	0.715228672	0.632588288	0.808665071	8.79E-08
hsa-miR-101-3p	0.70255517	0.584924782	0.843841435	0.000159325

Table S6. Survival related HR-DEmRNAs screened by univariate Cox analysis

id	HR	HR.95L	HR.95H	*P*-value
SAP30	1.425580195	1.1480045	1.770270842	0.001331095
SLC2A1	1.321323598	1.173594896	1.487647959	4.10E-06
ALDOA	1.302877164	1.112378599	1.525999249	0.001036363
ANXA2	1.274429412	1.064190168	1.526203094	0.008380938
STC2	1.263574017	1.119922891	1.425651094	0.000145076
GAPDH	1.247274221	1.019360878	1.526145466	0.031855296
NDRG1	1.220773069	1.072197449	1.389936983	0.002588579
PPFIA4	1.210447466	1.111127957	1.318644769	1.23E-05
EFNA3	1.193576264	1.057448879	1.347227585	0.004182391
PFKP	1.18035584	1.066476112	1.306395795	0.00135867
PGF	1.165433868	1.028662614	1.320390264	0.016232096
LOX	1.1491568	1.025371364	1.287885928	0.016810632
KDELR3	1.145858345	1.015316936	1.293183735	0.027364202
DPYSL4	1.137980992	1.054070788	1.22857094	0.000941545
KIF5A	1.137379752	1.04143547	1.242163089	0.004197617
ENO2	1.130077213	1.015733981	1.257292294	0.024652891
HK2	1.113928068	1.02561392	1.20984682	0.010464845
TKTL1	1.093492133	1.020370757	1.171853502	0.011372312
SLC2A5	1.067657378	1.003140329	1.136323846	0.039536942
DCN	0.928008504	0.869625035	0.990311626	0.024220045
PCK1	0.925320854	0.875063233	0.978464928	0.006448971
ALDOB	0.916118854	0.865984484	0.96915565	0.002280371
FBP1	0.880748873	0.811525341	0.955877209	0.002362277
DUSP1	0.865359146	0.756478405	0.989911207	0.035051838
ATF3	0.815535428	0.687661544	0.967188059	0.019110483

Table S7. The regulatory relationship between seven HR-DEmRNAs-os and six DEmiRNA-os

**miRNA**	**mRNA**
hsa-miR-7-5p	ENO2
hsa-miR-34a-5p	KDELR3
hsa-miR-34a-5p	PGF
**hsa-miR-101-3p**	PPFIA4
**hsa-miR-101-3p**	SAP30
hsa-miR-30d-5p	SAP30
hsa-miR-301a-3p	SLC2A1
**hsa-miR-101-3p**	TKTL1
**hsa-miR-195-5p**	TKTL1

Table S8. The regulatory relationship between six DEmiRNA-os and 17 DElncRNA-os

**lncRNA**	**miRNA**
SNHG1	**hsa-miR-101-3p**
AC005540.1	**hsa-miR-195-5p**
AC090772.3	**hsa-miR-195-5p**
CASC9	**hsa-miR-195-5p**
LINC00511	**hsa-miR-195-5p**
LINC01703	**hsa-miR-195-5p**
SNHG1	**hsa-miR-195-5p**
SNHG12	**hsa-miR-195-5p**
AP003469.4	hsa-miR-301a-3p
AC138150.2	hsa-miR-30d-5p
AL390728.6	hsa-miR-30d-5p
LINC01139	hsa-miR-30d-5p
AC073529.1	hsa-miR-34a-5p
AL365361.1	hsa-miR-34a-5p
MYLK-AS1	hsa-miR-34a-5p
AC012146.1	hsa-miR-7-5p
BACE1-AS	hsa-miR-7-5p
RHPN1-AS1	hsa-miR-7-5p
SNHG1	hsa-miR-7-5p

Table S11. Correlation between the expressions of seven HR-DEmRNA-os in hypoxia-associated ceRNA network and drug susceptibility of FDA-approved antitumor drugs in NCI-60 cancer cell line data

Gene	Drug	cor	*P*-value
ENO2	Docetaxel	-0.363931299	0.004257467
ENO2	Paclitaxel	-0.331330157	0.009710338
ENO2	Eribulin mesilate	-0.329886559	0.010052079
ENO2	Allopurinol	-0.321672869	0.012201806
ENO2	Vinorelbine	-0.30320039	0.018527508
ENO2	brigatinib	-0.292711897	0.023231814
ENO2	Carfilzomib	-0.292519726	0.023326648
ENO2	6-MERCAPTOPURINE	-0.274210683	0.033991762
ENO2	Celecoxib	-0.269913871	0.037010799
ENO2	Sonidegib	-0.264823653	0.040871658
ENO2	Vinblastine	-0.257065435	0.047390663
ENO2	Actinomycin D	-0.254271331	0.04993754
ENO2	Midostaurin	0.285390308	0.027083569
KDELR3	Valrubicin	-0.477126714	0.000116002
KDELR3	Cyclophosphamide	-0.469848508	0.00015214
KDELR3	Teniposide	-0.437839366	0.000467796
KDELR3	Palbociclib	-0.436460097	0.000489801

KDELR3	Daunorubicin	-0.43240181	0.000560107
KDELR3	Oxaliplatin	-0.431836951	0.000570586
KDELR3	Etoposide	-0.426820415	0.000671754
KDELR3	Pipobroman	-0.414999485	0.000977257
KDELR3	Nitrogen mustard	-0.403058727	0.001407912
KDELR3	Nelarabine	-0.397342296	0.001668991
KDELR3	Dexrazoxane	-0.392772785	0.00190802
KDELR3	Thiotepa	-0.384467139	0.002421962
KDELR3	Hydroxyurea	-0.379065008	0.002819224
KDELR3	Cytarabine	-0.377261066	0.002964233
KDELR3	Bendamustine	-0.376984292	0.002987058
KDELR3	Asparaginase	-0.369520533	0.00366447
KDELR3	DACARBAZINE	-0.362566167	0.004414587
KDELR3	Triethylenemelamine	-0.358055895	0.004970683
KDELR3	Ifosfamide	-0.355867997	0.005262003
KDELR3	Idarubicin	-0.355761857	0.005276509
KDELR3	Epirubicin	-0.349566785	0.006186576
KDELR3	Raloxifene	-0.347923617	0.006449935
KDELR3	Fluphenazine	-0.345051052	0.006933983
KDELR3	6-MERCAPTOPURINE	-0.340054031	0.007852011
KDELR3	Melphalan	-0.336779108	0.008509642
KDELR3	DECITABINE	-0.324233186	0.011492742
KDELR3	Mitoxantrone	-0.323854924	0.011595203
KDELR3	DIGOXIN	-0.323339919	0.011735973
KDELR3	Chlorambucil	-0.3201935	0.01262847
KDELR3	Dromostanolone Propionate	-0.319762249	0.012755242
KDELR3	Uracil mustard	-0.318715576	0.013067497
KDELR3	Eribulin mesilate	-0.318693512	0.013074149
KDELR3	Carfilzomib	-0.31750899	0.013435606
KDELR3	Docetaxel	-0.315550365	0.01405215
KDELR3	Vinblastine	-0.313038903	0.014878171
KDELR3	LDK-378	-0.308629304	0.016429443
KDELR3	Homoharringtonine	-0.306539629	0.017211623
KDELR3	Actinomycin D	-0.303576906	0.018374973
KDELR3	Doxorubicin	-0.302425062	0.018845014
KDELR3	Paclitaxel	-0.301607771	0.019184709
KDELR3	METHOTREXATE	-0.300183532	0.01978913
KDELR3	BMN-673	-0.296647618	0.021360092
KDELR3	6-Thioguanine	-0.289973032	0.024614634
KDELR3	Dexamethasone Decadron	-0.289448084	0.024887452
KDELR3	Raltitrexed	-0.288864184	0.025193899
KDELR3	Irinotecan	-0.288089814	0.02560521
KDELR3	Cladribine	-0.283981203	0.027883328
KDELR3	Crizotinib	-0.282920133	0.02849856

KDELR3	Fludarabine	-0.281437481	0.029377272
KDELR3	Fluorouracil	-0.274384724	0.033873932
KDELR3	Vincristine	-0.268661465	0.037931491
KDELR3	Isotretinoin	-0.259054727	0.045643005
KDELR3	Lomustine	-0.255995649	0.048352929
KDELR3	Cabozantinib	0.256012135	0.04833798
KDELR3	Encorafenib	0.258046675	0.046521884
KDELR3	Idelalisib	0.262500701	0.042740905
KDELR3	IPI-145	0.274032266	0.034112909
KDELR3	Abiraterone	0.277598648	0.031758417
KDELR3	Simvastatin	0.3103659	0.015802751
KDELR3	Zoledronate	0.314696918	0.014328314
KDELR3	Irofulven	0.316292216	0.013815824
PGF	Gefitinib	-0.286063225	0.026708499
PGF	Palbociclib	-0.28599749	0.026744945
PGF	Lapatinib	-0.282410865	0.028797865
PGF	Allopurinol	-0.257800736	0.0467384
PGF	Encorafenib	0.255806242	0.048524954
PGF	ARRY-162	0.270423714	0.036641338
PGF	Irofulven	0.27923532	0.030724224
PGF	Vemurafenib	0.320433069	0.012558515
PPFIA4	Dexrazoxane	-0.260150029	0.044703547
PPFIA4	IPI-145	0.276152087	0.032696527
PPFIA4	Abiraterone	0.29677486	0.021301779
PPFIA4	Idelalisib	0.297813681	0.020830733
SAP30	Erlotinib	-0.28213885	0.028958809
SAP30	Arsenic trioxide	0.263054711	0.042288829
SAP30	Lomustine	0.281176273	0.029534408
SAP30	Carmustine	0.33783721	0.00829211
SAP30	Nelarabine	0.375244089	0.003134181
SAP30	Ifosfamide	0.410794171	0.001113055
SLC2A1	Denileukin Diftitox Ontak	-0.341149431	0.007642167
SLC2A1	DIGOXIN	-0.282908223	0.028505529
SLC2A1	Arsenic trioxide	-0.260539819	0.044373074
SLC2A1	Bendamustine	-0.260393505	0.044496885
SLC2A1	Bortezomib	-0.257771797	0.04676393
SLC2A1	Ixazomib citrate	-0.257557848	0.046953039
SLC2A1	Dasatinib	0.257470285	0.047030616
SLC2A1	IPI-145	0.25944796	0.045303876
SLC2A1	Irofulven	0.265896991	0.040031031
SLC2A1	Simvastatin	0.286889008	0.026254205
TKTL1	6-Thioguanine	-0.500238155	4.70E-05
TKTL1	Parthenolide	-0.413381713	0.001027622
TKTL1	Allopurinol	-0.347091212	0.006587058

TKTL1	Pazopanib	-0.29664008	0.021363551
TKTL1	Bosutinib	-0.286142941	0.026664358
TKTL1	Lapatinib	-0.280386246	0.030013958
TKTL1	6-MERCAPTOPURINE	-0.267557154	0.038758975
TKTL1	TYROTHRICIN	0.273473193	0.034494855

Table S12. The expression correlation coefficient of miRNA-lncRNA pairs

miRNA	lncRNA	R	*P*-value
**hsa-miR-101-3p**	**SNHG1**	**-0.357**	**1.42E-12**
hsa-miR-195-5p	LINC01703	-0.186	3.16E-04
hsa-miR-195-5p	SNHG1	-0.151	3.61E-03
hsa-miR-195-5p	LINC00511	-0.147	4.48E-03
hsa-miR-195-5p	CASC9	-0.139	7.38E-03
hsa-miR-195-5p	AC005540.1	-0.127	1.43E-02
hsa-miR-195-5p	SNHG12	-0.095	6.74E-02
hsa-miR-195-5p	AC090772.3	-0.083	1.13E-01

Table S13. The expression correlation coefficient of mRNA-miRNA pairs

mRNA	miRNA	R	*P*-value
**PPFIA4**	**hsa-miR-101-3p**	**-0.384**	**1.76E-14**
**TKTL1**	**hsa-miR-101-3p**	**-0.151**	**3.49E-03**
**SAP30**	**hsa-miR-101-3p**	**-0.143**	**6.02E-03**
TKTL1	hsa-miR-195-5p	0.069	1.88E-01
